# Concurrent neuroimaging and neurostimulation reveals a causal role for dlPFC in coding of task-relevant information

**DOI:** 10.1038/s42003-021-02109-x

**Published:** 2021-05-17

**Authors:** Jade B. Jackson, Eva Feredoes, Anina N. Rich, Michael Lindner, Alexandra Woolgar

**Affiliations:** 1 MRC Cognition and Brain Sciences Unit, University of Cambridge, Cambridge, UK; 2 Perception in Action Research Centre, Department of Cognitive Science, Macquarie University, Sydney, NSW Australia; 3 School of Psychology and Clinical Language Sciences, University of Reading, Reading, UK

**Keywords:** Cognitive control, Attention

## Abstract

Dorsolateral prefrontal cortex (dlPFC) is proposed to drive brain-wide focus by biasing processing in favour of task-relevant information. A longstanding debate concerns whether this is achieved through enhancing processing of relevant information and/or by inhibiting irrelevant information. To address this, we applied transcranial magnetic stimulation (TMS) during fMRI, and tested for causal changes in information coding. Participants attended to one feature, whilst ignoring another feature, of a visual object. If dlPFC is necessary for facilitation, disruptive TMS should decrease coding of attended features. Conversely, if dlPFC is crucial for inhibition, TMS should increase coding of ignored features. Here, we show that TMS decreases coding of relevant information across frontoparietal cortex, and the impact is significantly stronger than any effect on irrelevant information, which is not statistically detectable. This provides causal evidence for a specific role of dlPFC in enhancing task-relevant representations and demonstrates the cognitive-neural insights possible with concurrent TMS-fMRI-MVPA.

## Abstract (web summary)

Jade Jackson et al. use fMRI concurrent with transcranial magnetic stimulation (TMS) in an attention task to evaluate whether the right dorsolateral prefrontal cortex (dlPFC) is involved in facilitation of relevant information, or suppression of irrelevant information. Their results suggest that the dlPFC is causally involved in processing relevant information in an attention task and highlight the utility of a dual fMRI-TMS approach.

## Introduction

A critical aspect of successful goal-directed behaviour is the ability to distinguish between information that is relevant for your current task and distracting irrelevant information. How does the brain achieve this selection? One prominent theory, termed the adaptive coding hypothesis^1–3^, posits that prefrontal neurons adjust their responses to preferentially code the information relevant for behaviour, which in turn modulates responses in specialised cortices^4,5^. In a similar vein, Miller and Cohen^6^ have long suggested that attention biases competing inputs in favour of relevant information (see also ref. ^7^). Evidence for these models stems from non-human primate (NHP) research where prefrontal neurons were shown to maintain task-relevant information in delayed-response tasks (see refs. ^8,9^) and flexibly encode the behavioural significance of visual stimuli, regardless of their physical properties^10–16^, while microstimulation work indicates causal top-down effects^17,18^. Recent observations indicate that prefrontal neurons can be driven by nonlinear combinations of multiple task features, referred to as mixed selectivity^19,20^, and that both relevant and irrelevant features, and task rules and decisions, can be coded by independent, dynamic patterns of activity across a neural population^21–24^. Together, these features align with a key role for the prefrontal cortex in selecting and integrating task-relevant information^3,21^.

In the human brain, a network referred to as the multiple-demand (MD) network^2^, has been shown to encode a range of task features^25^ with a strong preference for attended information over information that is irrelevant^26–30^. This network includes the dorsolateral prefrontal cortex (dlPFC), the anterior insula and frontal operculum (AI/FO), intraparietal sulcus (IPS) and the pre-supplementary motor area and adjacent anterior cingulate (ACC/pre-SMA). The MD network appears to be well-optimised for information selection, integration and exchange, with strong connectivity between the core regions of this network^31^ and task-dependent connections to other networks^32^. Of the regions of this network, the dlPFC is frequently theorised to be a likely candidate for top-down signals that influence neural activity according to behavioural relevance^6,33–37^, perhaps mediated by coherently oscillating neuronal assemblies^38–45^.

Despite this large body of work, causal evidence is still lacking. In the human brain particularly, we lack causal evidence relating dlPFC activity to the representation of information elsewhere. This follows since the majority of human work has used either neuroimaging, which alone is unable to draw causal links between network interactions, brain function and behaviour, or causal methods that do not incorporate neuroimaging or study of information coding. To overcome this limitation, in this study, we combined concurrent transcranial magnetic stimulation (TMS)—a causal method for intervening on neural activity—with functional magnetic resonance imaging (fMRI) and multivariate pattern analyses (MVPA). The disruptive effect of TMS allows for a test of causality, and the use of MVPA allows inference about the information coded in the system. Thus, our approach tests for the causal influences of dlPFC on information representation across the brain.

The combination of techniques also allowed us to address an open mechanistic question concerning the contribution of facilitatory and inhibitory mechanisms in information selection. Does prefrontal function primarily facilitate the representation of task-relevant information or contribute to suppressing task-irrelevant information? Some influential theories on selective attention have principally focused on the mechanisms that facilitate the processing of task-relevant information (e.g., adaptive coding hypothesis)^1^ with any suppression of irrelevant information resulting from local competition between inputs (e.g., biased competition)^7,46,47^. Early human fMRI work has supported a facilitatory account, for example, in a modified Stroop paradigm^48^, BOLD responses were increased in fusiform face area (FFA), under a high (incongruent trial follows an incongruent trial) compared to a low control condition (incongruent follows congruent), but only when the face stimuli served as targets, not as distractors. This was accompanied by increased functional coupling between FFA and dlPFC. Others, however, have emphasised direct inhibition mechanisms, such as the suppression of task-irrelevant brain regions mediated by alpha-band oscillations^49–52^. For example, occipital alpha power is typically higher contralateral to the unattended side of space than contralateral to the attended side^53–56^, with alpha-power increases linked to the proactive suppression of distractors^57^, and decreases associated with increased spike activity and better task performance^58^. These alpha modulations may be driven by prefrontal areas^41^. On the other hand, it has been argued that the data typically taken as evidence for suppression are equally compatible with an account of selection through signal enhancement^59^. It may be the case, for example, that the observed effects reflect secondary inhibition related to facilitation of the relevant inputs.

This is difficult to untangle without intervening on the system, but a previous concurrent TMS-fMRI study demonstrates the logic for how we might contrast facilitatory and inhibitory effects. Feredoes et al.^60^ stimulated right dlPFC while participants remembered a target and ignored a distractor. They showed that dlPFC TMS modulated BOLD signals specifically in posterior brain areas corresponding to the current memory targets (e.g., FFA for faces), rather than the distractor items (e.g., parahippocampal place area, for houses), suggesting that control is exerted primarily through modulating processing of task-relevant items. Here, we used a similar logic but went a step further in examining the effect of TMS on the representation of task-relevant and -irrelevant information, which, using MVPA, we could separate out even within single brain regions. This is important because modulations in the overall signal may not reflect changes in the representation of information, and because it allowed us to ask about facilitation and inhibition in frontoparietal regions that might reasonably be expected to respond to multiple different aspects of stimuli. Thus, we aimed to provide a strong test of the contribution of facilitatory versus inhibitory mechanisms in selection by establishing the causal impact of dlPFC on the representation of this information elsewhere in the brain.

In this study, participants selectively attended to one (relevant) feature of a visual object, such as its colour, and ignored another (irrelevant) feature of the same object, such as its shape, whilst we applied a short train of TMS to the right dlPFC. Right dlPFC was selected as the stimulation target because TMS to this region of interest (ROI) has been shown to modulate activation in a task requiring selective attention^60^. We tested the following hypotheses: the primary role of the right dlPFC is to (1) upregulate relevant information, (2) downregulate irrelevant information or (3) both upregulate relevant and downregulate irrelevant information. If the right dlPFC normally enhances coding of attended information, then disrupting it with TMS should decrease coding of attended stimulus features across the MD network (disruption to the system) and visual cortices (top-down modulation). Conversely, if the right dlPFC suppresses irrelevant information, then disrupting this function should increase coding of the unattended feature. If right dlPFC plays both roles, then TMS should both decrease relevant and increase irrelevant information coding. Another hypothesis (4) is that the right dlPFC may play a general role in supporting all information processing (no specific role in attentional selection). Under this alternative, TMS would decrease both relevant and irrelevant information coding. A final hypothesis (5) is that the right dlPFC has no role in supporting information processing, in which case we would expect no change in information coding with TMS. We tested these predictions in the MD regions, to test network function^2,3^, and in visual cortices (lateral occipital complex (LOC), V4, early visual cortex) and across the whole brain (using a roaming searchlight), to assess top-down modulation^4,37^. The results supported hypothesis 1 with Active TMS reducing coding of relevant information in the MD regions, but having no detectable effect on coding of irrelevant information. The data provide causal evidence that the dlPFC biases processing elsewhere in the brain by selectively facilitating coding of task-relevant information.

## Results

**Fig. 1:**
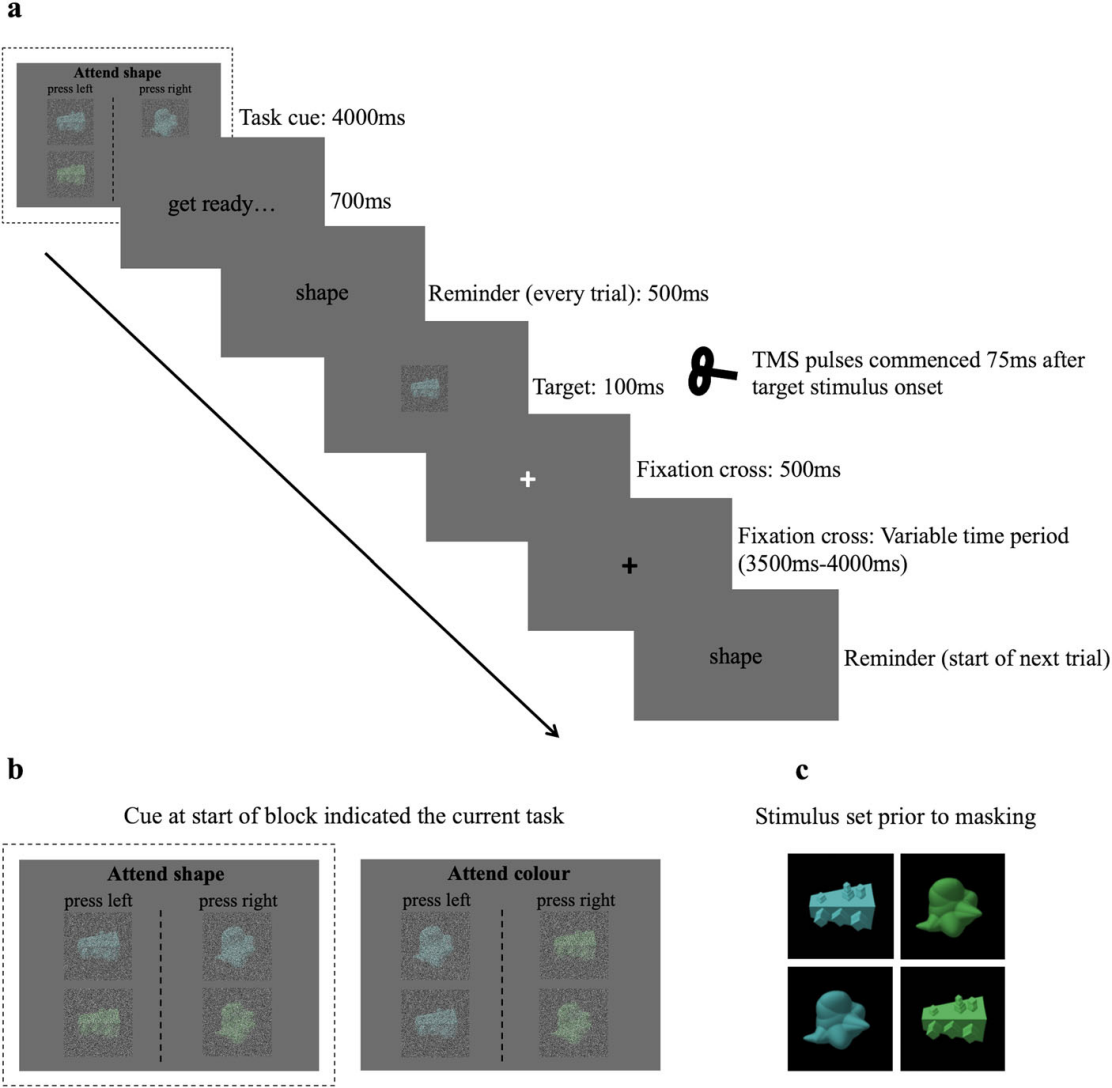
Concurrent TMS-fMRI task. On each trial, participants saw a target object that they had to categorise according to either its form (angular/curvilinear) or colour (green/blue) in alternating blocks (**a**). A picture cue at the start of each block indicated the current task (form or colour; **b**). On each trial, a cue reminded participants of the current task (500 ms) followed by the object to categorise (100 ms), during which they received a train of 3 TMS pulses (13 Hz, high or low intensity, onset 75 ms after stimulus). Participants were instructed to respond before the white cross (500 ms) turned black. If participants responded within 500 ms, the white cross turned black for the remainder of the 500 ms and was in either case followed by a black cross for a jittered interval of 3500–4000 ms. In the example shown, participants are cued to attend to shape and the correct response would be the left button. The stimulus–response mappings for the two dimensions meant that for some stimuli, their shape and colour required the same button response (congruent) and for other stimuli, their shape called for one button and its colour called for the other button (incongruent). The trial depicted in this figure is a congruent trial as the correct button response for its shape is left, and its blue colour would also have indicated the left button during the colour task. In (**c**), the four presented objects are displayed prior to masking for illustration purposes.

**Fig. 2:**
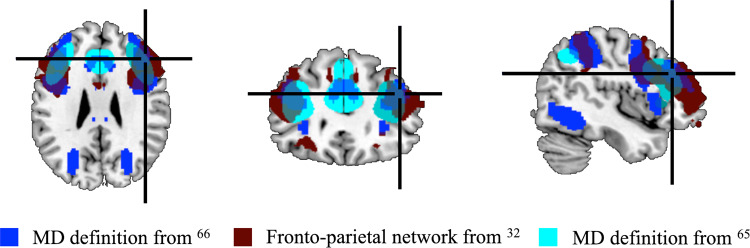
Reference coordinate for TMS target site in right dlPFC. Functional activation and connectivity maps from previous studies (as in legend) were used to select MNI152 coordinates [44 31 28] as a group-level reference for right dlPFC target (cross hair). After deforming to native space, we defined the target stimulation site on an individual-participant basis using the peak activation from individual-participant functional localiser data that was within 14 mm of this reference point.

We combined TMS with fMRI and used MVPA to examine the causal influence of right dlPFC activity on information coding in the brain. Participants attended to and reported one feature of a novel object (e.g., it’s colour) whilst ignoring an irrelevant feature (e.g., its form) in alternating task blocks (Fig. 1). We adopted a TMS protocol where participants received a train of 3 pulses, 75 ms following stimulus onset, at a frequency of 13 Hz. The train was delivered to the right dlPFC (Fig. 2) on every trial at either high-intensity (110% motor threshold (MT), Active condition) or low-intensity TMS (40% MT, Control condition).

### Behavioural data

As an indirect measure of the relative contribution of the relevant and irrelevant stimulus dimensions to behaviour, we compared performance for stimuli in which colour and form mapped onto the same button-press response (congruent) to those where the two stimulus dimensions indicated different button-press responses (incongruent). We further asked whether this congruency effect was modulated by TMS and checked that there was no modulation of these effects by task (colour or form). To do so, we analysed the data using three-way repeated-measures ANOVAs, with factors Congruency (Congruent, Incongruent), TMS (Control, Active) and Feature (Colour, Form).

**Fig. 3:**
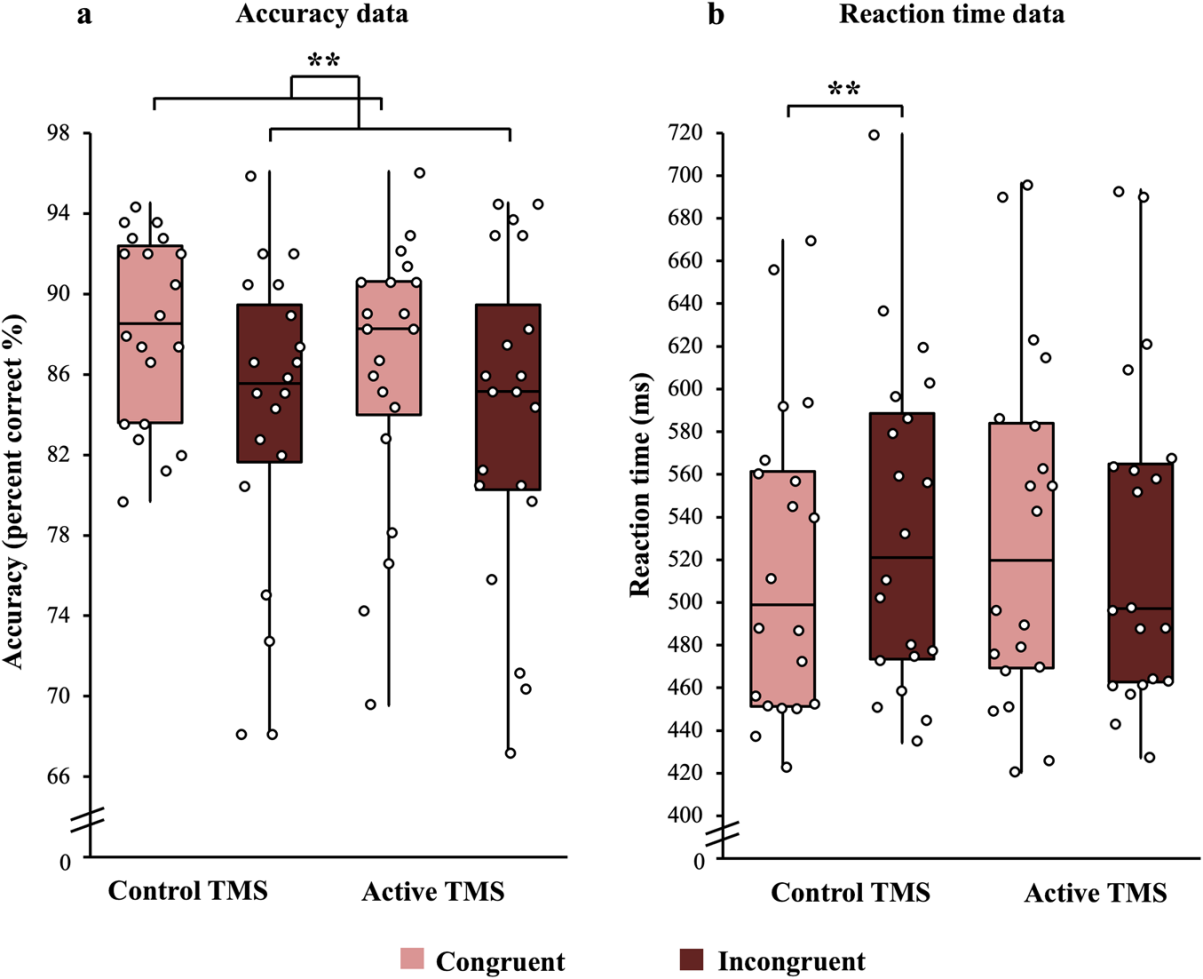
The effect of TMS on accuracy and reaction time data. Box plot summary of accuracy (**a**) and reaction time (**b**) data depicting the minimum and maximum values, the lower and upper quartiles and the median scores. Individual data points are indicated by open circles. Lighter-coloured bars depict congruent trials, and darker-coloured bars depict incongruent trials. Accuracy data (**a**) showed a main effect of congruency (no interaction). RT data (**b**, correct trials only) revealed that participants were faster in congruent trials than incongruent trials under the Control TMS condition but showed no evidence of a congruency effect under Active TMS (significant interaction). **P < 0.01. *N* = 20 participants.

There was a main effect of Congruency on participants’ accuracy (Fig. 3a, bar chart representation in Supplementary Fig. 1) (*F*(1,19) = 11.58, *M*_diff_ = 3.28 (95% CI: 1.26, 5.29), *P* = 0.003, *η*^*2*^_*p*_ = 0.38), reflecting more accurate performance on congruent (87.2%) relative to incongruent (84%) trials overall. This classic congruency effect confirmed that the experimental design induced conflict between the relevant and irrelevant stimulus dimensions. The size of this congruency effect was not significantly affected by TMS (*P* = 0.39, BF_10_ = 0.35) and, despite a numeric trend for reduced accuracy under Active TMS, no other main effects or interactions were significant (all *P*s > 0.23, all BF_10_ < 0.51).

For reaction time (RT) data (correct trials only, Fig. 3b, bar chart representation in Supplementary Fig. 1), there was a significant interaction between TMS and Congruency (*F*(1,19) = 4.78, *P* = 0.04, *η*^*2*^_*p*_ = 0.2). Post hoc paired *t* tests showed that participants were significantly faster on congruent (517 ms) than incongruent trials (534 ms; *t*(19) = 4.43, *M*_diff_ = 16.9 (95% CI: 8.91, 24.9), *P* < 0.001, *d* = −0.99) in the Control TMS condition, but in the Active condition, there was no significant difference between congruent (532 ms) and incongruent (528 ms) trials (*t*(19) = 0.46, *M*_diff_ = 3.57 (95% CI: −12.6, 19.8), *P* = 0.65, *d* = 0.1), with Bayesian analysis indicating evidence for the null hypothesis of no effect (BF_10_ = 0.26). No other main effects or interactions were significant (all *P*s > 0.11, all BF_10_ < 1.01). These data suggest that TMS modulated the size of the congruency effect on RT, with Active TMS tending to reduce the benefit of congruent trials seen under Control conditions. Note, however, that in order to make the task difficult, participants were given a limited time window in which to respond (i.e., were rushed), which makes the RT data more complex to interpret. As such, we do not derive strong conclusions from the RT data.

### The effect of TMS on overall activation levels

**Fig. 4:**
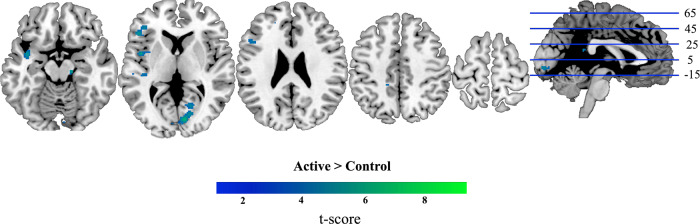
Brain regions showing a larger BOLD response under Active than Control TMS (univariate contrast). We examined differences in overall BOLD response under Active (high intensity) and Control (low intensity) TMS using a mass-univariate whole-brain approach. We modelled Active and Control trials separately, and contrasted BOLD responses at the second level with paired *t* tests at each voxel. These results were thresholded at *P* < 0.0001 (FWE correction *P* < 0.05 at cluster level). Coordinates of peaks are given in Supplementary Table 1. Group-level analysis for the converse analysis (Control > Active) showed no significant clusters of activation. *N* = 20 participants. Axial slices are depicted in neurological convention (i.e., the left hemisphere is on the left-hand side of the image).

We conducted a whole-brain mass-univariate analysis to examine whether overall activation levels in different brain regions were affected by Active TMS to right dlPFC (Fig. 4 and Supplementary Table 1). Relative to Control TMS, Active TMS increased BOLD response in clusters of voxels in and around the left temporal cortices (Heschl’s gyrus, superior temporal gyrus), likely reflecting the difference in auditory stimulation between the two TMS conditions. We also observed increased BOLD in clusters in/around the frontoparietal MD network (right ACC, left dlPFC) and visual cortices (left extrastriate cortex, right primary visual cortex), under Active TMS, demonstrating long-range effects of dlPFC stimulation. The reverse contrast (Control > Active) showed no significant clusters.

### The effect of TMS on information coding

#### Unstimulated MD regions

**Fig. 5:**
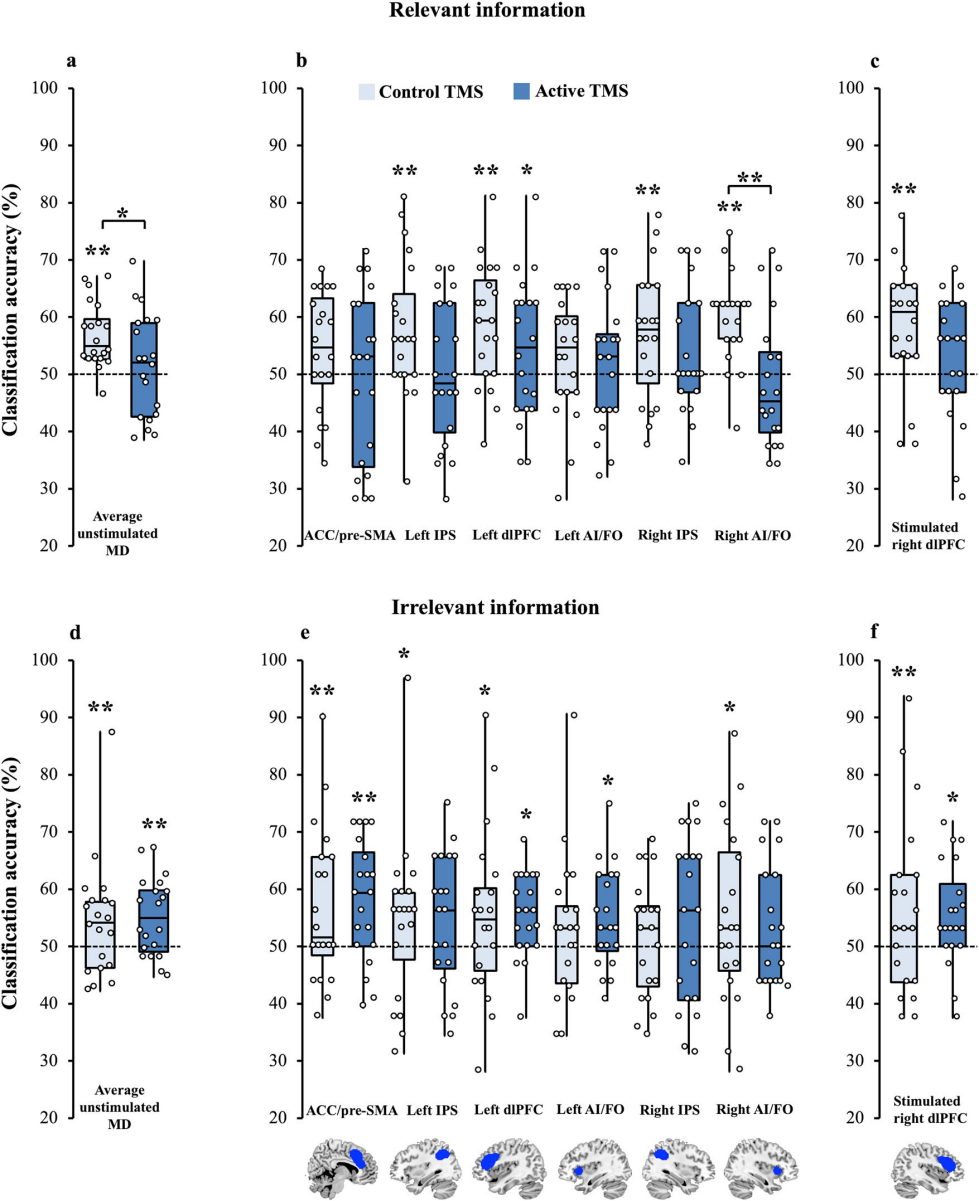
Coding of relevant and irrelevant information in MD regions under Control and Active TMS conditions. Box plot summary depicting minimum and maximum values, lower and upper quartiles and median scores. Individual data points indicated by open circles. Lighter-coloured bars depict coding under control TMS, and darker-coloured bars depict coding under Active TMS trials. **a**–**c** show coding of relevant information (e.g., colour during the colour task) under Control and Active conditions, collapsed across feature (colour, form). **d**–**f** show coding of irrelevant information (e.g., colour during the form task) under Control and Active conditions, also collapsed across feature. All bars represent coding of identical stimulus information, variation in the strength of coding is driven by TMS intensity and whether the information was relevant for the participant’s current task. Due to outliers (>3 SD from the condition mean), we performed a log transformation on the unstimulated MD region data before statistical testing. The data displayed are in the untransformed form prior to log transformation. An ANOVA on the unstimulated MD regions (**a**, **d**) showed a significant TMS × relevancy interaction. TMS reduced coding of relevant features in unstimulated MD regions, but did not modulate coding of irrelevant information (BF_10_ = 0.24). The ANOVA for right dlPFC (factors: TMS, Feature and Relevancy; **c**, **f**) showed no significant main effects or interactions. The significance markings for individual bars indicate whether coding was significantly greater than chance in each condition separately (permutation test). *P < 0.05. In this figure only, ** is equal to *P* < 0.008 (to correct for multiple comparisons in 6 unstimulated MD regions). *N* = 20 participants.

Next, we turned to our main question, which was whether TMS to right dlPFC affected information coding, and whether this modulation differed for information that was relevant versus irrelevant to the current task. For this, we examined multivariate decoding of object colour (green vs. blue) and form (angular vs. curvilinear) when relevant (e.g., green vs. blue in the colour task) and irrelevant (e.g., green vs. blue in the form task) under the two separate TMS conditions (Control and Active). First, we examined information coding in the unstimulated MD network. Figure 5 shows the classification accuracies for the unstimulated MD regions for relevant (Fig. 5a, average MD; Fig. 5b, individual MD ROIs) and irrelevant (Fig. 5d, average MD; Fig. 5e, individual MD ROIs) information separately (bar chart representation in Supplementary Fig. 2). We entered these classification accuracies into an ANOVA with factors TMS, Feature, Relevancy and Region. There was a significant interaction between TMS and Relevancy (*F*(1,19) = 5.04, *P* = 0.037, *η*^*2*^_*p*_ = 0.21) indicating that that TMS had a differential impact on coding of relevant and irrelevant information. Post hoc paired *t* tests revealed that relevant information coding was significantly reduced under Active TMS (51.4%) compared to Control TMS (56.8%; *t*(19) = 2.39, *M*_diff_ = 5.42 (95% CI: 0.67, 10.2), *P* = 0.03, *d* = 0.53). However, for irrelevant information coding, there was no significant difference between Control (54.2%) and Active (55%) TMS conditions (*t*(19) = 0.28, *M*_diff_ = 0.8 (95% CI: 5.04, −6.64), *P* = 0.78, *d* = −0.06). Bayesian analysis indicated evidence for the null hypothesis of no effect of TMS on irrelevant information coding (BF_10_ = 0.24). The ANOVA also revealed a main effect of Feature (*F*(1,19) = 6.35, *M*_diff_ = 5.04 (95% CI: 0.85, 9.21), *P* = 0.02, *η*^*2*^_*p*_ = 0.25) driven by overall stronger coding of colour (56.9%) compared to form (51.8%), but this baseline effect did not interact with any other factors. No other main effects (all *P*s > 0.29, all BF_10_ < 0.62) or interactions (all *P*s > 0.13, all BF_10_ < 0.34, task × relevancy interaction *P* = 0.09 and BF_10_ = 7.61) were significant. Specifically, BF_10_ for Region and its interactions were all less than 0.13, indicating evidence that the pattern of results was similar across all MD regions.

The significant effect of TMS on decoding of relevant information is only interpretable if coding in one or more of the TMS conditions is significantly above chance. Therefore, we compared classification accuracies against chance, using a two-step permutation test. We found that relevant information was significantly coded under Control TMS (permutation test, *P* < 0.001) but was not under Active TMS (by permutation, *P* = 0.35, BF_10_ = 0.28). Therefore, the significant effect of TMS on relevant information coding is interpretable: TMS to right dlPFC significantly reduced coding of relevant stimulus information in the rest of the MD system. The interaction further specifies that this effect was larger than any effect on irrelevant information coding, which was not detected.

#### Right dlPFC (stimulated site)

The right dlPFC was the target for stimulation, so we analysed it separately from the rest of the MD system (Fig. 5c, f). It is typical not to see effects at the stimulation site with our setup^61^. One reason for this is that the TMS coil may shield this part of the cortex from MR excitation and readout. Although the trend appeared to be in the same direction as the rest of the MD system, our ANOVA (factors TMS, Feature and Relevancy) detected no significant main effects or interactions (all *P*s > 0.22, all BF_10_ < 0.57).

#### Visual cortices

**Fig. 6:**
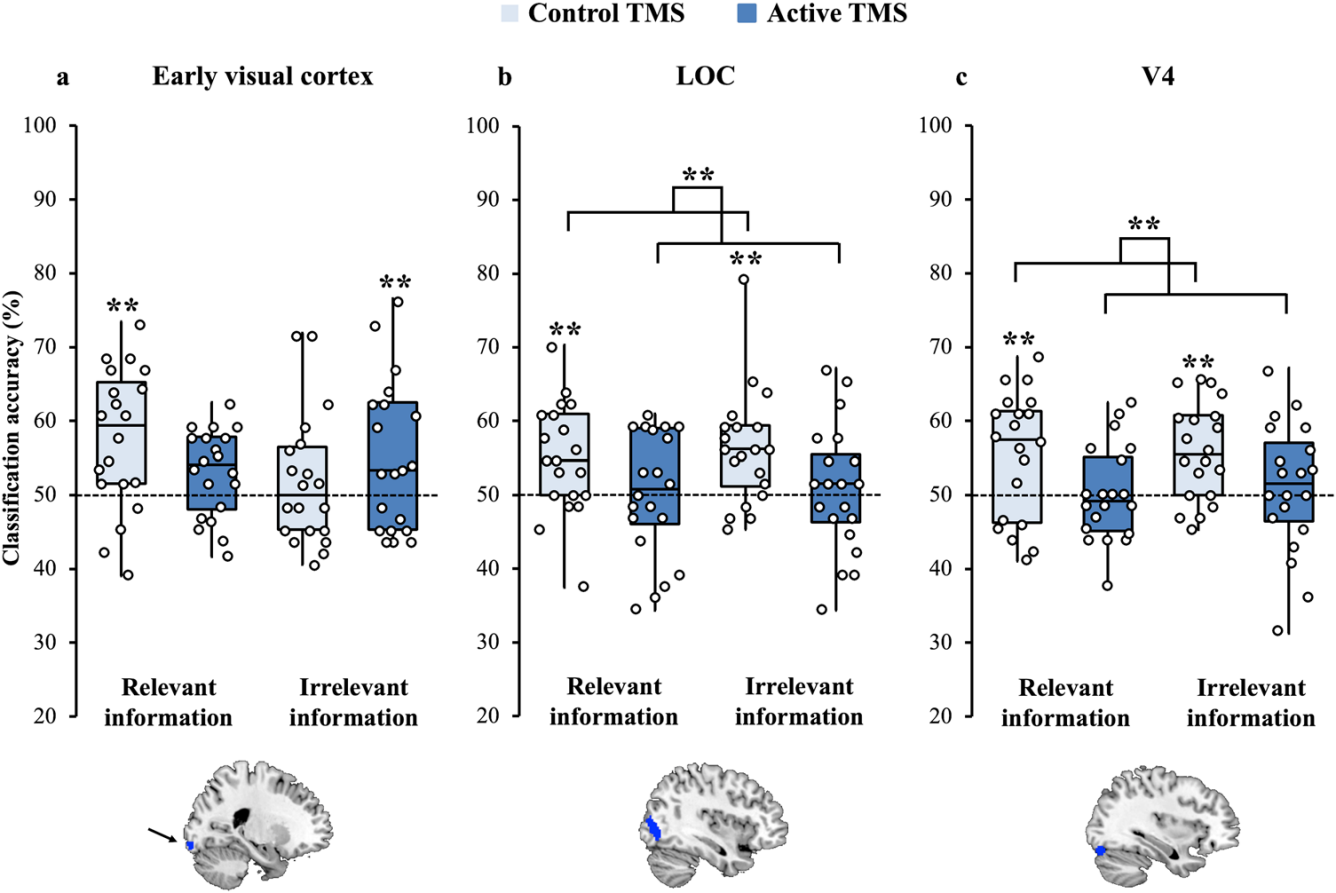
Coding in visual ROIs under Control and Active TMS. Box plot summary depicting the minimum and maximum values, the lower and upper quartiles and the median scores. Individual data points indicated by open circles. Lighter-coloured bars depict coding under control TMS, and darker-coloured bars depict coding under Active TMS trials. **a** Early visual cortex (central visual field). This ROI was derived from individual-participant localiser data and defined as the region stimulated by visual information at fixation (encompassing the same area of the central visual field as the objects in the main experimental task) minus visual information outside fixation. There were no significant main effects or interactions. **b** Lateral occipital complex (LOC). This ROI was derived from localiser data as the region more active for viewing of whole objects over scrambled objects. There was stronger coding under the Control TMS condition compared to the Active condition modulated by a Feature × TMS interaction reflecting a stronger effect of TMS on colour than form coding. **c** V4. This ROI was derived from coordinates from the literature^120^ and transformed into native space for each participant. There was again the main effect of TMS modulated by a Feature × TMS interaction reflecting a stronger effect of TMS on colour than form coding. Significance markings for individual bars indicate whether coding was significantly greater than chance in each condition separately (by permutation). *P < 0.05; **P < 0.01. *N* = 20 participants.

Next, we examined top-down effects in the visual cortex. The trend in the early visual cortex region (defined from individual-subject localiser data, Fig. 6a, bar chart representation in Supplementary Fig. 3) was for a decrease in relevant and an increase in irrelevant information coding, but the statistical analysis did not show any significant main effects or interactions (all *P*s > 0.07, all BF_10_ < 1.43).

In LOC (defined from individual-subject localiser data, Fig. 6b, bar chart representation in Supplementary Fig. 3), we observed a different pattern. Here, there was a significant main effect of *TMS* (*F*(1,19) = 10.1, *M*_diff_ = 5.15 (95% CI: 1.75, 8.55), *P* = 0.005, *η*^*2*^_*p*_ = 0.35) with stronger coding for Control than for Active TMS, without a TMS × Relevancy interaction (*F*(1,19) = 0.03, *P* = 0.86, *η*^*2*^_*p*_ = 0.002, BF_10_ = 0.21). These data align with a general top-down effect of dlPFC activity on all stimulus processing, irrespective of relevancy. However, this main effect was modulated by a significant interaction between TMS and Feature (*F*(1,19) = 6.29, *P* = 0.02, *η*^*2*^_*p*_ = 0.25), which reflected a stronger effect of TMS on colour coding (Control 57.9%, Active 48.1%, post hoc paired *t* test *t*(1,19) = 4.22, *M*_diff_ = 9.87 (95% CI: 4.97, 14.7), *P* < 0.0001, *d* = 0.94) than on form coding (Control 53.4%, Active 52.9%, *t*(1,19) = 0.17, *M*_diff_ = 0.44 (95% CI: −5.05, 5.92), *P* = 0.87, *d* = 0.04, BF_10_ = 0.24). This may reflect the overall tendency, seen also in the MD regions, for colour to be coded more strongly than form in our task. No other main effects or interactions were significant (all *P*s > 0.47, BF_10_ < 0.45).

In colour-responsive cortex (V4, defined by coordinates from the literature, Fig. 6c, bar chart representation in Supplementary Fig. 3), we saw a similar pattern. There was again a significant main effect of *TMS* (*F*(1,19) = 9.72, *M*_diff_ = 5.18 (95% CI: 1.71, 8.67), *P* = 0.006, *η*^*2*^_*p*_ = 0.34) with stronger coding for Control than for Active TMS, and no TMS × Relevancy interaction (*F*(1,19) = 0.01, *P* = 0.91, *η*^*2*^_*p*_ = 0.001, BF_10_ = 0.24). There was also, again, a significant TMS × Feature interaction (*F*(1,19) = 5.61, *P* = 0.03, *η*^*2*^_*p*_ = 0.23), reflecting a stronger effect of TMS on colour than on form. Post hoc paired *t* tests showed a significant effect of TMS on colour coding (Control 59.6%, Active 50.2%, post hoc paired *t* test (*t*(1,19) = 4.17, *M*_diff_ = 9.37 (95% CI: 4.67, 14.1), *P* = 0.001, *d* = 0.93) and no effect on form coding (Control 51.9%, Active 50.9%, *t*(1,19) = 0.38, *M*_diff_ = 0.99 (95% CI: −4.44, 6.43), *P* = 0.71, *d* = 0.09, BF_10_ = 0.25). No other main effects or interactions reached significance (all *P*s > 0.06, all BF_10_ < 1.04).

Overall, right dlPFC-TMS had a significant effect on information coding in higher visual cortex ROIs (LOC, V4), but there was no evidence that this effect differed for relevant and irrelevant information processing.

#### Searchlight analysis

We conducted an exploratory analysis to check for additional regions in which coding was affected by TMS to the right dlPFC by performing decoding analyses across the whole brain using a roaming searchlight^62^. The advantage of this approach is that is it free from a priori spatial hypotheses, meaning we can potentially identify additional regions missed by the ROI approach, but it has a lack of power relative to ROI analyses, given a large number of comparisons it entails.

**Fig. 7:**
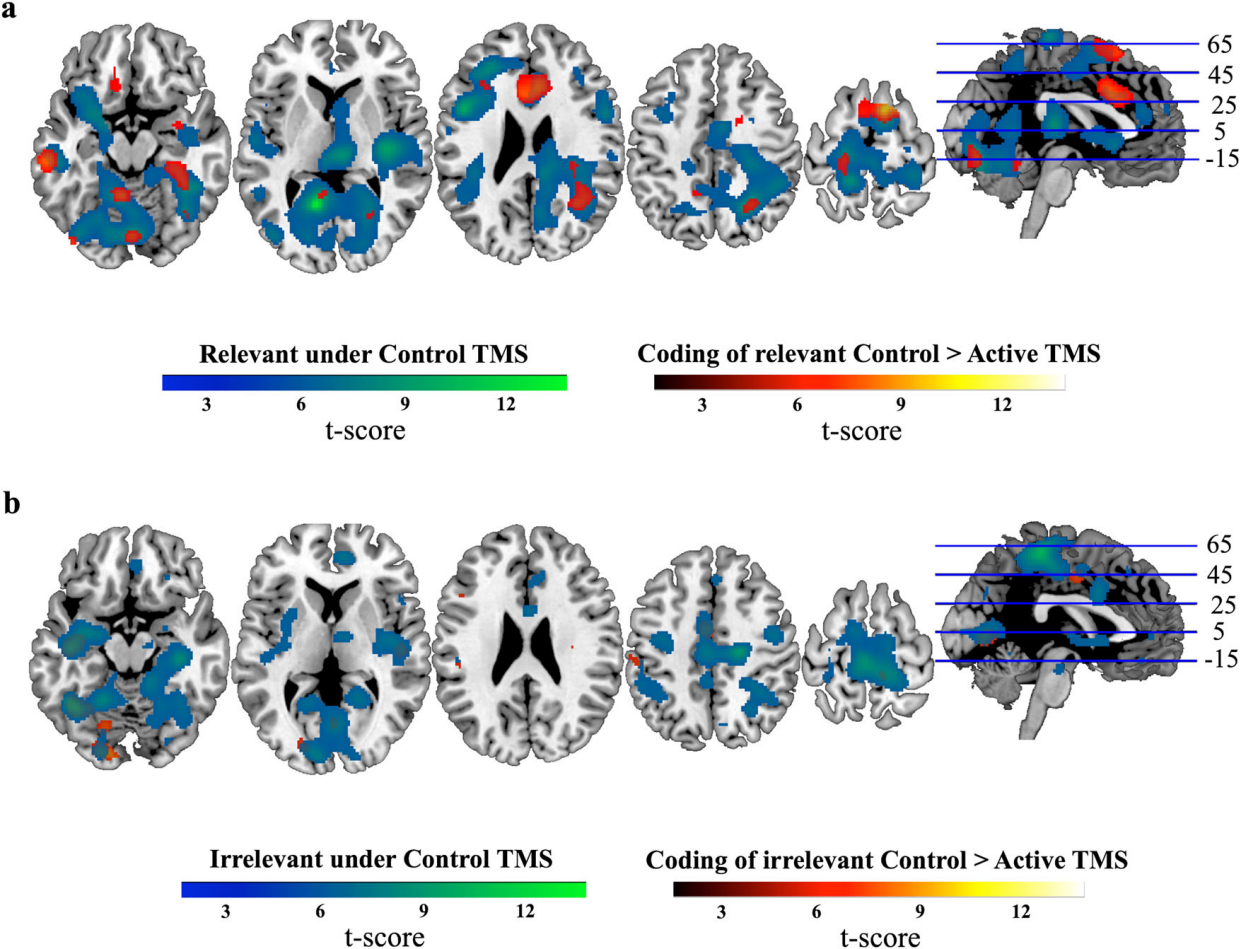
Coding of relevant and irrelevant information assessed with a roaming searchlight. Whole-brain maps indicate where patterns of activation in the local neighbourhood (10-mm sphere) discriminated relevant information (**a**) or irrelevant information (**b**) under Control TMS (blue–green), and where coding was significantly reduced from Control to Active TMS (red–yellow). No other contrasts showed significant clusters and are not depicted. The results were thresholded at *P* < 0.0001 (FWE correction at the cluster level, *P* < 0.05). Coordinates of peak decoding are given in Supplementary Tables 2 and 3. *N* = 20 participants. Axial slices are depicted in neurological convention (i.e., the left hemisphere is on the left-hand side of the image).

We performed searchlights comparing coding to chance in each of the relevancy and TMS conditions separately, and then compared these maps to one another. Under Control TMS, significant coding of relevant information was seen across the brain, including in/around the MD network and visual cortices (Fig. 7a and Supplementary Table 2). For irrelevant coding, five large clusters survived correction at the cortical and subcortical level under Control TMS (Fig. 7b and Supplementary Table 2). For both conditions, no clusters survived correction under Active TMS.

The direct comparison of these maps revealed stronger coding of relevant information under Control compared to Active TMS in six clusters, including in and around the ACC, left dlPFC, as well as in occipital, frontal and temporal cortices (Supplementary Table 3 and Fig. 7a). There were no significant clusters for the reverse contrast (Active > Control). For irrelevant information (Control > Active), there were several significant clusters, including in occipital and temporal cortices (Supplementary Table 3 and Fig. 7b). There were no significant clusters for the reverse contrast (Active > Control).

**Fig. 8:**
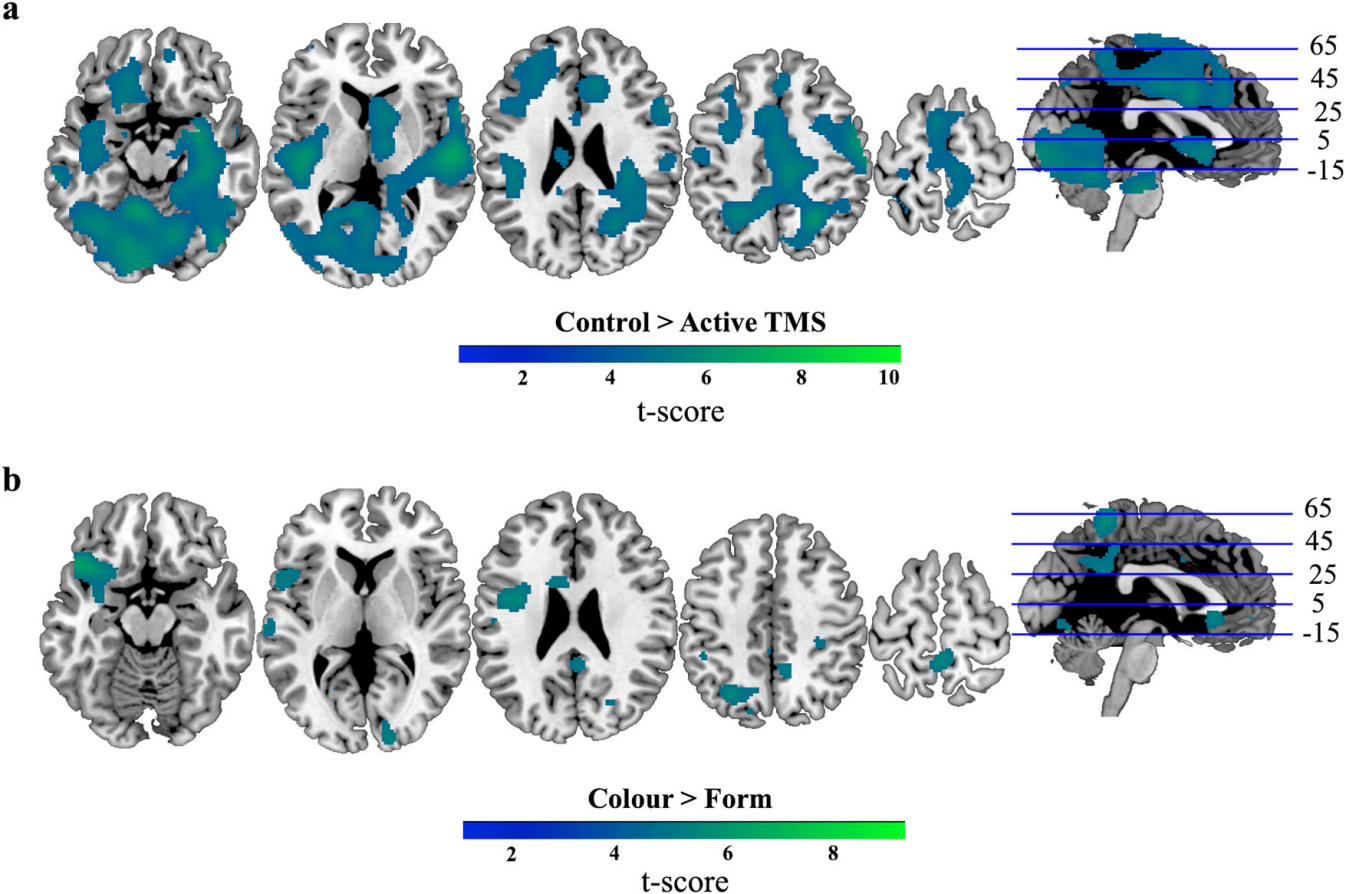
Information coding assessed with a roaming searchlight as revealed by a three-way repeated-measures ANOVA. Whole-brain maps indicate where patterns of activation in the local neighbourhood (10-mm sphere) discriminated information more strongly under Control > Active TMS (**a**), and for decoding of Colour > Form (**b**), revealed as main effects in the three-way repeated-measures ANOVA. The results were thresholded at *P* < 0.0001 (FWE correction of *P *< 0.05 at cluster level). *N* = 20 participants. Axial slices are depicted in neurological convention (i.e., the left hemisphere is on the left-hand side of the image).

Finally, for comparison of all our conditions, we analysed the data with a repeated-measures ANOVA (factors: TMS, Feature, Relevancy). This showed no significant clusters for either the three-way, or the two-way interactions. Significant clusters were observed in the lateral occipital cortex extending across visual cortices, cerebellum, post-/pre-central gyrus, superior temporal gyrus and frontal pole, and in the frontal medial cortex extending into cingulate gyrus, for the main effect of TMS (Control > Active, Fig. 8a), indicating a drop of information under Active TMS. For the main effect of Feature (Colour > Form, Fig. 8b), significant clusters were observed in the frontal orbital cortex extending to the precentral gyrus, and in the temporal pole, precuneus, supramarginal gyrus, occipital pole and superior temporal and posterior cingulate gyrus, reflecting stronger coding of colour than shape, as in the ROI analyses. There were no significant clusters for the main effect of Relevancy.

The results of these exploratory analyses indicate that right dlPFC activity supports coding of task-related information across a range of brain regions. However, in concordance with the predefined ROI analyses, there was no evidence for a release from suppression following stimulation to the right dlPFC. Qualitatively, the effect of TMS on relevant information appears to be larger in the resultant maps; however, this interaction did not reach significance as it did in the predefined MD network.

## Discussion

We used multivariate analyses of concurrent TMS-fMRI data to causally examine the role of right dlPFC in supporting attentional processing in the brain. In a selective attention task, perturbing right dlPFC with TMS affected information coding in the MD network, visual cortices and other brain areas, commensurate with a role for the right dlPFC in supporting brain-wide information processing. We tested the following hypotheses: (1) dlPFC is causally involved in upregulating relevant information, (2) dlPFC is causally involved in downregulating irrelevant information, (3) dlPFC function is both to upregulate relevant information and to downregulate irrelevant information, (4) dlPFC plays a general role in supporting all information processing or (5) dlPFC has no role in supporting information processing. The data provided evidence for hypothesis 1, suggesting that the role of the dlPFC was primarily in supporting the coding of task-relevant information processing. Active TMS impaired MD coding of relevant visual object information (object form or colour when needed for the task), but had no detectable effect on MD coding of the identical visual object information when it was not needed for the task (e.g., an object’s colour when participants were reporting the object’s form). Moreover, parametric statistics confirmed that the effect of TMS on relevant information coding in the MD system was significantly larger than any effect on irrelevant information, and Bayesian analyses suggested evidence for the absence of an effect on irrelevant information. These data advance understanding of causal mechanisms supporting the prioritisation of task-relevant information in the human brain.

The dlPFC is thought to be crucial for goal-directive behaviour. It is part of a circuit of frontal and parietal brain regions, referred to as the MD regions^2,3,31^, task-positive network^63^ or frontoparietal control system^64^. These areas are known to be engaged by a wide variety of task demands (see refs. ^65,66^) and have been posited to play a fundamental role in executive function and cognitive control^5,67^. NHP has demonstrated that many neurons in these areas exhibit mixed selectivity, encoding individual task variables in distinct multidimensional subspaces that evolve over time^23,68^. Both relevant and irrelevant sensory data have been shown to be represented^21,69^ although there appears to be a preference towards task-relevant information^10,11,13–16^, a result mirrored in human neuroimaging data^26–30^. Exactly how these dynamic neuronal assemblies provide feedback and bias downstream regions is as yet unclear, although it has been suggested that different information could be carried in distinct frequency bands^70^, and that selective representation could support preferential coding in other brain regions^4,6,37^. For example, Baldauf and Desimone^71^ combined magnetoencephalography (MEG) and fMRI to show that the inferior frontal junction appears to direct object-based attentional inputs to the inferior–temporal cortex. In addition, using multivariate analysis in MEG and/or electroencephalography, several authors^72–75^ have reported that patterns of information coding in occipital brain regions can be Granger-caused by information coding happening earlier in time in frontal regions. More broadly, strong connectivity between core MD regions^31^ and task-dependent connections to other networks^32^ is highly suggestive of a system optimised for information exchange and integration, perhaps mediated by coherently oscillating neuronal assemblies^76,77^. However, these previous data are not sufficient to establish a causal role. In parallel, findings from offline TMS^78^ and patients with chronic focal lesions (see refs. ^79,80^), suggest a causal role for the MD network in attention and higher cognition, but do not establish the neural mechanisms by which it is achieved. The current work bridges this gap by demonstrating a causal role for dlPFC in supporting the representation of task-relevant information in the frontoparietal cortex and other, anatomically distributed, brain regions.

The data further suggest that the mechanism by which the right dlPFC supported selective processing in our task was primarily through facilitating processing of task-relevant information, rather than through suppressing irrelevant information. The dlPFC has previously been suggested to be involved in inhibitory mechanisms (see refs. ^35,37,81–83^) and to drive oscillatory dynamics that may coordinate both inhibitory and facilitatory signals to bias other brain structures^36,77^. Had we interfered with such a process with TMS, we would have expected to see a release from inhibition for distracting (suppressed) information. This would have been seen as an increase in coding of the irrelevant information. Such a result would also be predicted by the biased-competition framework^4,81^, particularly for the visual cortex, based on reduced local competition from the downregulated relevant information. Our data, however, fail to find evidence for either of these accounts, with our Bayesian analyses indicating evidence for no effect of TMS on irrelevant information coding (MD regions), and a reduction in coding across relevancy conditions in the higher visual cortex (LOC and V4 ROIs) and searchlight clusters in/around cingulate gyrus, visual cortices, superior temporal gyrus, cerebellum and post-/pre-central gyrus. No regions showed a TMS-related increase in irrelevant information coding in either the ROI or searchlight analyses. Thus, the present work provides causal evidence that dlPFC activity facilitates the representation of relevant information in the related network with no evidence for suppressive mechanisms.

The extent to which we can generalise a finding from one type of task to another is, of course, unknown. It may be possible to see evidence of inhibitory effects with more data, or with a technique with the higher temporal resolution, or with different TMS parameters, or in a task that places the different sources of information in more direct competition. It also does not rule out suppressive mechanisms in general, which might, for example, be revealed with TMS to a different brain region. However, the interaction between the effect of TMS and relevancy in the unstimulated MD network specifies that, in this task, the role of right dlPFC was primarily facilitating task-relevant, rather than inhibiting irrelevant, information processing. These findings support major accounts of higher cognitive functions (see refs. ^2,6,33,84^) that posit dlPFC as mediating control by facilitating processing of attended information.

In contrast to the clear effects on decoding, TMS had little effect on participant behaviour, with no measurable impact on accuracy and, if anything, a diminished congruency effect in the RT data. Limited behavioural effects are not uncommon in TMS-fMRI paradigms^60,85,86^. It may be that behavioural measures are less sensitive to the small perturbation that TMS causes or reflect resilience in the neural system that we did not capture here. Along the same lines, it seems likely that the diminished congruency effect in RT (to the extent that it can be interpreted, given that participants responded under time pressure) reflects changed neural processes that were not captured by our neural analyses. In particular, our design was optimised to distinguish between the neural representation of relevant and irrelevant stimulus dimensions. By contrast, in behaviour, it is challenging to separate the contribution of each dimension, since every response, presumably, reflects some combination of the two. The significant behavioural congruency effect demonstrates that the two dimensions conflicted at some level of processing, but cannot specify the level at which this conflict occurred or was resolved (e.g., stimulus processing, response processing^87–89^). This underscores the complexity in relating TMS-induced changes in BOLD to changes in cognition and behaviour^90,91^ and reinforces the intuition that behaviour is more than a simple combination of the strength of stimulus representation measured with MVPA. Future TMS-fMRI work may be able to relate behavioural and neural decoding data more directly using simpler designs where both neural data and behaviour can be analysed for the same information, or, for example, by interrogating the information content on behavioural errors^92^.

Note that in the present report, we applied TMS to the right hemisphere, and thus can only draw conclusions about the causal involvement of right dlPFC. Previous decoding work has rarely reported any difference in attentional modulation of information coding between left and right dlPFC^29,93,94^, which are highly connected^31^ and form part of the frontoparietal MD network^2,3^. However, recent work has shown that while the MD network is frequently recruited as a whole, the precise pattern of recruitment can differ from task to task^31,95^. As we could only stimulate one location, we based our selection on prior evidence that TMS to the right dlPFC modulates activation in a task requiring selective attention^60^. Future work targeting left dlPFC would be needed to assess possible hemispheric distinctions. Moreover, from the current data, we cannot rule out that similar results would be seen with disruption to another region of the MD network, or indeed another brain region altogether^96^: our results suggest causal involvement of the right dlPFC in selection of task-relevant information, but do not specify that this is the only region with this role. Future work, targeting different brain regions, is needed to determine the specificity of the observed effect.

The present findings showed a disruptive effect of stimulation, with decreased coding of task information under the Active condition. The relationship of these data to the univariate contrast showing that overall BOLD was increased under the Active condition is unclear. Numerous factors could have led to differences between the Active and Control stimulation conditions here. For example, there was increased activation in several auditory regions under Active TMS, presumably reflecting differences in acoustic noise between the Active and Control conditions. Moreover, the overall BOLD level is derived from a combination of inhibitory and excitatory signals; thus, it is problematic to interpret an increase in overall BOLD as indexing a purely facilitatory or disruptive effect^97,98^. Differences between multivariate and univariate results are not uncommon^99^ and, in this case, multivariate analyses may be more informative, as they provide us with evidence relating to the integrity of task-related information processing.

The TMS protocol applied in the present study consisted of three pulses at 13 Hz. These parameters were chosen based on previous work that demonstrated inhibitory effects on behaviour with a similar protocol^100,101^. A recent meta-analysis also confirmed that higher frequencies (>10 Hz) tend to be disruptive in attention-based paradigms^102^ with some dependency on the frequency of ongoing neuronal oscillations in the targeted region^103,104^. However, the exact mechanism of action by TMS on neuronal processing is still under debate^91^. There are several proposals, one being that TMS induces disorganised activity in the stimulated region (i.e., induces neural noise^90^) and another being that TMS suppresses neural activity, decreasing the signal rather than adding to the noise^105^. Recent work using concurrent stimulation and recording suggests a complex picture involving periods of both inhibition and excitation following parietal stimulation at 110% MT^106^. These effects corresponded to impaired behaviour and were absent at low (60% MT) amplitude. This pattern of excitation–inhibition may lead to a loss of entropy and disruption in ongoing computations^96^. These data provide more detail about the neurophysiological effects of TMS, and support our choice of comparing stimulation at high and low intensity, but substantially more work is needed to be certain of the neural effect expected from the large parameter space of TMS protocols, particularly for repetitive protocols.

Concurrent TMS-fMRI experiments are both technically challenging and associated with high attrition rates. Accordingly, the present report had a final sample size of 20, despite the initial recruitment of 31 participants. Another potential issue is that there is limited information from the region under the stimulation site, because the TMS coil may have shielded the targeted cortex from MR excitation and readout. This means we cannot independently confirm that we stimulated our precise target of interest. As neuronavigation was performed outside of the scanner room and the coil orientation and its position occasionally had to be adjusted slightly to accommodate the MR coil, TMS targeting was likely imperfect. However, given that the magnetic field of TMS is somewhat diffuse^107^, and that the area of dlPFC activated as part of the MD network is actually relatively large (e.g., from Duncan and Owens’^65^ template definition of right dlPFC, the average ROI size across participants was 19.4cm^3^), any small coil targeting adjustments is unlikely to have a noticeable effect. For future work, we are encouraged by recent developments of in-MR and in-bore neuronavigation technology (Localite) and dedicated TMS-fMRI high-sensitivity coil arrays^108^, which give excellent resolution directly under the coil (~5% BOLD signal change^109^) compared to previous setups with ~0.5% signal change directly underneath the coil (e.g., ^110^). These advances will increase the accuracy and confidence in target ROI stimulation in the future.

In this study, we observed modulated coding of task-related information across the frontoparietal network, as well as long-range effects on other brain networks. The effect of TMS on the coding of relevant information appeared to be more widespread than the modulation of irrelevant information, but the searchlight analysis did not reveal the same TMS*Relevancy interaction that was observed in the predefined MD network. This may be due to a relative lack of power in the searchlight analysis or may reflect a more general top-down effect of dlPFC-TMS that affects representation of both types of task information. Areas where coding of relevant information was reduced under Active TMS (and relevant information was coded above chance under the Control condition) included occipital cortices (peak in lingual gyrus), cerebellum, LOC (extending to precuneus) and the MD regions (ACC/pre-SMA and left dlPFC). Comparatively, regions where coding of irrelevant information was reduced under Active TMS (and irrelevant information was coded above chance under the Control condition) included visual areas (occipital fusiform, LOC), temporal cortices, precuneus and thalamus. The data emphasise that perturbation of right dlPFC causes top-down modulation of information in distant regions, underscoring the long-range connectivity associated with the frontoparietal cortex^111^.

With these data, we examined the way in which the right dlPFC exerts attentional control, by separating facilitation of task-relevant information from the inhibition of task-irrelevant information. The findings provided causal support only for facilitatory mechanisms, with perturbation of right dlPFC with TMS specifically disrupting coding of relevant, as opposed to the equivalent irrelevant, information, in the unstimulated frontoparietal network. Exploratory analyses suggested that disruption to dlPFC also affected coding of both relevant and irrelevant information in discrete distal regions, but there was no evidence for a release from suppression of irrelevant information anywhere in the brain. The data provide strong causal evidence in support of major theories implicating the prefrontal cortex in executive control and suggest that the primary mechanism for control by right dlPFC is in biasing processing towards information that is relevant to our behaviour.

## Methods

### Participants

Thirty-one healthy volunteers signed up for the experiment. However, four participants did not pass the TMS screening requirements, and seven participants did not complete the second scanning session, so their data are not included. The final group consisted of twenty participants (15 females, 5 males; mean age = 21.6 years, SD = 3.36). This sample size was the maximum possible given the available funding, time constraints, attrition rate, the requirement to scan each participant twice and the technical challenges involved in acquiring fMRI data concurrent with TMS. A post hoc power analysis estimated that for our main analysis of interest, we achieved 62% power to detect a TMS × Relevancy interaction at *d* = 0.05 for the reported effect size of partial *η*^*2*^ = 0.2. All participants were right-handed with normal or corrected-to-normal vision and no history of neurological or psychiatric disorder. Participants gave written informed consent and received £30.00. The experiment was approved by the University of Reading Research Ethics Committee and all ethical regulations were followed.

### Stimuli

Stimuli were abstract novel objects created using custom scripts, following^112^. The stimulus set consisted of four objects (see Fig. 1c), which were either blue (RGB: 98 179 180) or green (RGB: 95 171 96) and were one of two novel shapes (angular or curvilinear, referred to elsewhere as “cuby” and “smoothy”^27,112^). To increase perceptual difficulty, we superimposed a noise filter in Adobe Photoshop CC (2014), which applies random pixels to the picture. The colour values used for the noise creation were distributed on a bell-shaped (i.e., Gaussian) curve. We applied the monochromatic filter that allows for a degree of colour preservation within the image, because the filter applies pixels that differ only in tone, and not in actual colour, from the original image. In separate blocks of trials, participants reported either object colour (blue or green), or object form (angular or curvilinear). Thus, the visual feature that was relevant varied between blocks. We controlled stimulus presentation with a PC running the Psychophysics Toolbox-3 package^113^ in MATLAB (Mathworks).

### Overall procedure

Participants attended two sessions separated by 2–8 days. In Session 1, we determined the participant’s resting MT and familiarised them with the sensation of TMS. They also had a structural MRI scan and completed three functional localiser tasks in the MR scanner to determine the stimulation site and ROIs for further analysis. In Session 2, participants completed the main task in the scanner, with concurrent TMS. The same TMS machine and coil were used in Session 1 for determining individual MTs as in Session 2 for the main experiment. Below we outline the procedures in detail.

#### Session 1: Motor threshold

For each participant, we first acquired their resting MT outside of the scanner. For this, we determined the minimum intensity at which a single pulse through the TMS coil, positioned over the hand area of the primary motor cortex, produced a visible twitch in the abductor pollicis brevis when at rest, in five of ten successive pulses. Individuals’ MTs determined stimulation intensity for that participant in Session 2. Stimulation intensity in the scanner in Session 2 was pseudorandomly varied over trials within a block at either 110% (Active stimulation) or 40% (Control stimulation) of the individual participant’s MT. After their MT was determined, participants were given instructions and completed the structural scan and localiser tasks.

#### Session 1: Right dlPFC localiser

We used a functional localiser alongside activation maps from previous studies (see section: Selection of the stimulation target) to determine our target site in dlPFC. The functional localiser was a modified version of the main experimental task. On each trial, participants saw an object that they had to categorise according to either its form (angular/curvilinear) or colour (green/blue) in alternating blocks. Within each block, there was a mini block of eight congruent followed by eight incongruent trials (or vice versa, for the localiser, congruent and incongruent trials were blocked to give maximal power for analysis). Congruent trials comprised objects where the response button for the irrelevant dimension was congruent with the required response for the relevant dimension. Conversely, the incongruent blocks consisted of objects where the response for the irrelevant dimension was incongruent with the required response for the relevant dimension. For example, if a participant was instructed to respond “left” for blue and “right” for green when reporting colour, and “left” for angular and “right” for curvilinear when reporting form, then a green angular object would be an incongruent trial (i.e., “right” button response in the colour task, “left” button response in form task) and a blue angular object would be congruent. Rest blocks were also included (black cross at fixation: 16 s) in-between the colour and form alternating task blocks. Task order (colour/form) and block type (congruent/incongruent) were counterbalanced across participants.

Participants practised outside the scanner for a minimum of six blocks and until they achieved >70% performance. For the first two practice blocks and after each stimulus presentation, participants viewed a black cross (2000 ms) during which time they were told that their responses were recorded. Following the first 2 blocks and to mimic the main experimental task, immediately following object presentation, a white cross appeared for 500 ms followed by a black cross for 1100 ms. If participants responded within 500 ms, the white cross turned black for the remainder of the 500 ms. The white cross served to add time pressure, as participants were told that their responses were only recorded during the white cross period (responses were, however, still recorded during the black cross period). In the first two practice blocks, participants received feedback on every trial. Following this, they only received feedback (percent correct) at the end of the block.

In the scanner, participants completed two localiser runs (6 min each). Within each run, there were 18 blocks of each condition: congruent (16.8 s, 8 trials/block), incongruent (16.8 s, 8 trials/block) and rest (16.8 s). Participants received feedback (percent correct) at the end of every block. In the second run, the button response mapping (the left- and right-hand button response associated with each colour/object) was switched to mimic the procedure of the main task (below). The response-mapping switch was included to dissociate activity associated with participant’s motor responses from that with the stimulus features. The button response mapping order was counterbalanced across participants.

We defined dlPFC as the region close to the inferior frontal sulcus that responded more strongly on incongruent than congruent trials (see “Selection of the stimulation target” below). We reasoned that this activity would reflect the processes required to selectively attend to relevant over irrelevant information.

#### Session 1: Early visual cortex localiser

This localiser was designed to identify the region stimulated by visual information at fixation (encompassing the same area of the central visual field as the objects in the main experimental task). Participants viewed a small white circle at the centre of the screen that was visible across all blocks. Rest blocks (16 s) consisted only of the circle presentation. The stimulus blocks (16 s) included either a flickering checkerboard presented only at fixation (the spatial extent matched the objects in the main task) or a checkerboard filling the entire field of view leaving only a blank grey screen in place of the fixation checkerboard. Participants were required to press a button when the central circle on the screen flashed green (0.1 s) to encourage attention to the screen. The EPI time was 8 min.

#### Session 1: LOC localiser

The LOC localiser was designed to functionally identify object-sensitive cortex. Participants viewed centrally located intact and scrambled versions of black and white objects in 16.8-s blocks of 16 trials (1100 ms/trial), whilst attending to a central fixation cross. Participants indicated via a button response when the fixation cross changed from black to blue. There were 21 blocks consisting of alternating blocks of whole objects, scrambled objects and rest blocks (order counterbalanced across participants). The EPI time was 6.25 min.

#### Session 2: Overview

In Session 2, we implemented the Brainsight infrared frameless stereotaxy neuronavigational system (Rogue Research Inc.) to navigate to each participant’s individual target stimulation location, and marked the site on their scalp. Following this, participants practised the main task outside the scanner and then completed eight runs in the scanner with simultaneous online TMS on every trial.

#### Session 2: Selection of the stimulation target

We defined the target stimulation site based on individual subject data cross-referenced to functional activation (those regions showing activation for a wide range of tasks from refs. ^65,66^) and connectivity data (the frontoparietal network from ref. ^32^) from the literature (Fig. 2).

We calculated two contrasts from our dlPFC localiser: (1) incongruent > congruent and (2) incongruent + congruent > rest. We then derived a comparison sphere centred on MNI coordinates [44 31 28] at the intersection of the activation and connectivity maps and deformed it into individual subject native space. If the dlPFC localiser peak activation (see section “Right dlPFC for stimulation” under “ROI definition” for contrast details) from contrast (1) was <14 mm from the centre of the comparison sphere, then the central coordinate of peak activation was selected for that participant as their stimulation target (*n* = 12; furthest individual peak coordinate was 13.08 mm). If contrast (1) showed no activation (minimum initial threshold of *P* < 0.001, uncorrected) within the range of the comparison sphere, then we compared activation for contrast (2) applying the same procedure (*n* = 5). For the remaining 3 participants, there were no clusters of activation from either contrast within 14 mm of the comparison sphere, so the central point of the sphere was chosen as the target.

#### Session 2: Neuronavigation

We used the Brainsight neuronavigational system to guide coil placement to the individual stimulation target coordinates, using standard neuronavigation routines. The target location was marked on the scalp for stimulation inside the scanner. The final position of the TMS coil was adjusted in the scanner to fit within the MR head coil and to be comfortable for participants. The TMS coil was oriented with the handle pointing posteriorly with respect to the participant’s head, and roughly parallel to the midline, to target the frontoparietal network as opposed to the default mode network^114^. For some participants, adjustments to the coil orientation were necessary to ensure no part of the TMS coil was touching the head coil. Orientation adjustments were also made, if necessary, to minimise any discomfort produced by the stimulation, which can sometimes be the case for the dlPFC scalp location.

#### Session 2: Concurrent TMS-fMRI (main experimental task)

Before entering the scanner, participants again practised the task for at least 6 blocks without TMS. For this and the main task, the task was blocked (colour, form). Within each block, congruent and incongruent trials were presented pseudo-randomly (not in mini-blocks as they had been for the localiser). There were no rest blocks and the timings were slowed down, relative to the task localiser (see Fig. 1a for task design). This was to ensure that the time between the TMS trains met with safety recommendations^115^. During the practice, participants received feedback after each trial for the first two blocks as well as feedback (percent correct) at the end of the block. For the last four blocks of practice, and in the scanner, participants only received feedback at the end of the block. Participants repeated the last four blocks of practice trials until they scored >70% correct.

In the scanner, participants completed 8 runs (6.3 min each) of the task with concurrent TMS. For this, an MR-compatible TMS figure-8 stimulating coil (MRI B90 II, MagVenture, Farum, Denmark) was held firmly in position inside the MR head coil, by a custom-made non-ferromagnetic coil holder. The cable of the TMS coil passed through the back of the scanner and out through a waveguide and connected to the TMS machine (Magpro X100, MagVenture, Farum, Denmark) located in the MR control room.

In each scanning run, participants completed one block of the colour task and one block of the form task. The block started with a picture cue (4000 ms, Fig. 1b) indicating the current task context and response mapping. On each trial, participants first saw a written cue reminding them of the current task (“colour” or “shape”, 500 ms) followed by the target object (100 ms). Participants received a train of three TMS pulses: the first pulse at 75 ms after target stimulus onset, and then the remaining pulses separated by 75 ms (13 Hz). A TMS protocol of three pulses at 13 Hz was chosen here as inhibitory effects on behaviour have been observed in previous work with a similar protocol^100,101^. Pulses were triggered by a MATLAB (Mathworks) script on a PC, which also received pulse timings from the MR machine and controlled the visual stimulus delivery to the projector. Pulses were delivered to coincide with the readout phase of slice acquisition. They were timed so that the artefact caused by each TMS pulse would affect only one MRI slice and would occur at a different slice of each volume^116^. The affected slice was later discarded and interpolated over (see below). The train of pulses was delivered at 110% (Active) or 40% (Control) of participant’s MT, as in our previous work^60^. The intensity of stimulation was varied pseudo-randomly over trials. The choice of low intensity as the control condition allowed for the two TMS conditions to be interleaved in the scanner on a trial-by-trial basis, ensuring that the upcoming level of stimulation was unpredictable to participants. Low-intensity TMS controls for the non-specific effects of TMS, and targets the same ROI as the main stimulation site, and is therefore a more conservative control than, for example, a no-TMS comparison (following previous concurrent TMS-fMRI work^60,85,86,117^). There were a total of 1536 pulses across all of the runs, complying with published safety limits for TMS stimulation^115^. Immediately following object presentation (100 ms), a white cross appeared for 500 ms followed by a black cross for a variable time period (3500–4000 ms). If participants responded within 500 ms, the white cross turned black for the remainder of the 500 ms. The participants were told that their responses were only recorded during the white cross period (responses were actually still recorded during the black cross period). Participants received feedback at the end of each block. After the first four runs, the button-response mapping for both colour and form was swapped (e.g., if the button response for green had been the left-hand button response, green would now require a right-hand button response). Mappings were swapped for both colour and form, so the congruency of response for any given object did not change.

### Data acquisition

FMRI data were collected for both scanning sessions using a Siemens Magnetom Trio 3 T whole-body MRI scanner at Centre for Integrative Neuroscience and Neurodynamics, Reading University, Reading, UK.

#### Session 1: Localisers

We used a sequential ascending T2*-weighted EPI acquisition sequence with the following parameters: acquisition time 2080 ms, echo time 30 ms. 60 oblique axial slices with a slice thickness of 3.0 mm and a 0.70-mm inter-slice gap, in-plane resolution 3.0 × 3.0 mm, matrix 64 × 64, field of view 256 mm and flip angle 78°. T1-weighted MPRAGE structural images were also acquired for all participants (slice thickness 1.0 mm, resolution 1.0 × 1.0 mm).

#### Session 2: TMS-fMRI experiment

We used a sequential ascending T2*-weighted EPI acquisition sequence with the following parameters: acquisition time 2450 ms, echo time 30 ms, 35 oblique axial slices with a slice thickness of 3.0 mm and a 0.70-mm inter-slice gap, in-plane resolution 3.0 × 3.0 mm, matrix 64 × 64, field of view 256 mm and flip angle 90°; 50% phase oversampling in the phase-encoding direction to shift any Nyquist ghost artefact, due to the presence of the TMS coil, to outside the volume of interest.

### Preprocessing

#### Session 1: Localisers

MRI data were preprocessed using SPM 5 (Wellcome Department of Imaging Neuroscience, www.fil.ion.ucl.ac.uk/spm) in MatLab 2013b. Functional MRI data were converted from DICOM to NIFTI format, spatially realigned to the first functional scan and slice timing corrected, and structural images were co-registered to the mean EPI. EPIs were smoothed (8-mm FWHM Gaussian kernel), and in all cases, the data were high-pass filtered (128 s). Structural scans were additionally normalised to the T1 template of SPM5 (Wellcome Department of Imaging Neuroscience, London, UK, www.fil.ion.ucl.ac.uk), using SPM5’s segment and normalise routine. This was done to derive the individual participant normalisation parameters needed for transformation of ROIs into native space and TMS target definition, and to normalise the searchlight classification maps derived in native space.

#### Session 2: TMS-fMRI experiment

Following conversion of the functional data from DICOM to NIFTI format, we removed the slices that were affected by TMS pulses (one slice per pulse). We first identified slices with a signal magnitude of >1.5 SD from the run mean and visually inspected them for the presence of the TMS artefact. These slices were replaced by temporal interpolation of the signal values of the same slice from the preceding and succeeding volumes (following ref. ^60^). Next, we manually removed and interpolated over any remaining slices that were acquired during TMS pulse delivery, identifying them based on timing and visual inspection. This was necessary because, depending on the affected slice, the Control TMS condition did not always produce deviations >1.5 SD from the mean. We ensured that the same number of slices were removed and interpolated over in the Active and Control TMS conditions. Aside from this slice removal step for Session 2 data, preprocessing followed the same steps as Session 1. EPIs from Session 2 were smoothed slightly (4-mm FWHM Gaussian kernel) to improve signal-to-noise ratio for multivariate analyses, and were smoothed separately with a larger smoothing kernel for univariate analyses (8-mm FWHM Gaussian kernel).

### ROI definition

#### Right dlPFC for stimulation

Both right and left dlPFC have been implicated in selective attention^29,93,94^. Here, as we could only stimulate one side, we chose the right dlPFC based on previous research demonstrating TMS to this region modulates activation in a task requiring selective attention^60^. We used the multiple- regression approach of SPM5 to estimate values corresponding to the congruent, incongruent and rest conditions in the dlPFC localiser data. Blocks were modelled using a box car function lasting 16.8 s convolved with the haemodynamic response of SPM5. The run mean was included in the model as a covariate of no interest. Whole-brain analyses (paired *t* tests) compared blood-oxygen-level-dependent (BOLD) responses across the following conditions: [incongruent – congruent], which was the congruency effect, and ((incongruent +  congruent) – rest), which was the task effect. The resulting map was thresholded such that there was at least one cluster in the right hemisphere with a minimum size of 20 voxels. We used this lenient thresholding as the purpose was to identify an ROI for target stimulation. The peak of one of these clusters was chosen as the target stimulation site as explained above in the section: “Session 2: Selection of the stimulation target”.

#### Early visual cortex

Blocks of early visual cortex localiser data were modelled using a box car function lasting 16 s convolved with the haemodynamic response of SPM5. The run mean was included in the model as a covariate of no interest. We contrasted BOLD responses with paired t-tests in the two conditions (fixation checkerboard minus outside-fixation checkerboard). The resulting maps were thresholded so that there were two clusters, of a minimum size of 20 voxels, in early visual cortex. The mean size of early visual cortex across participants was 84.3 voxels (S.D. 9.13).

#### LOC

We estimated values pertaining to the whole and scrambled object conditions in the LOC localiser. Blocks were modelled using a box car function lasting 16 s convolved with the haemodynamic response of SPM5. The run mean was included in the model as a covariate of no interest. Mass univariate paired *t* tests compared voxelwise BOLD response in the two conditions (whole objects minus scrambled objects). For each subject separately, the resulting maps were thresholded such that there was at least one cluster with a minimum size of 20 voxels in LOC in each hemisphere (close to coordinates ([−44 −67 −10], [41 −67 −11], and [−41 −78 −2], [39 −73 −3] , from previous studies^118,119^). We used this lenient thresholding as the purpose was to identify an ROI for MVPA analysis. The mean size of LOC across participants was 150.8 voxels (S.D. 85.8).

#### V4 (colour-responsive cortex)

The V4 ROI was defined from the previous data^120^, centred on coordinates [−32 −82 −20; left hemisphere] and [32 −82 −20; right hemisphere]. The mean size of V4 across participants was 56.1 voxels (S.D. 5.23).

#### MD network

We used the template MD ROI definition from our previous work^26–28,93,94^, which was derived from a meta-analysis of activity associated with a diverse set of cognitive demands^65^. We opted to use a template definition of the MD network because recent work has indicated that there is no particular benefit to using individual subject functional localisers to define these regions for multivariate analysis^121^, whereas the template approach made it easy to compile the data across the group. Note that when we analysed fMRI responses in the right dlPFC, we used this template definition, and not the individual-subject data from the dlPFC localiser, which was only used to define the TMS target. The average size in voxels across participants for each region was ACC-pre SMA: 792.0 (S.D. 65.8), IPS: 787.8 (S.D. 26.1), left dlPFC: 676.8 (S.D. 43.8), right dlPFC: 685.6 (S.D. 49.5) and AI/FO: 359.1 (S.D. 11.0).

### Statistics and reproducibility

#### Univariate contrast: the effect of TMS on overall activation levels

We examined differences in the overall BOLD response under Control (low intensity) and Active (high intensity) TMS using a mass-univariate whole-brain approach. A general linear model (GLM) was estimated for each participant using the realigned, slice-time corrected and smoothed normalised EPI images (8-mm FWHM Gaussian kernel) from Session 2. We modelled Control and Active trials separately and contrasted BOLD responses at the second (random effects across subjects) level with one-tailed paired *t* tests at each voxel (for both Control > Active and Active > Control). The results were thresholded at *P* < 0.0001 (cluster-level family-wise error (FWE) correction for multiple comparisons). All coordinates are reported in MNI152 space.

#### MVPA: First-level model for concurrent TMS-fMRI task

To obtain estimated activation patterns for MVPA, we estimated a GLM for each participant with SPM5 using the preprocessed images from Session 2. We estimated the activity associated with the two colours and two forms of the objects, using correct trials only. Each trial contributed to the estimation of two beta values: the relevant feature (green or blue in the colour task, and angular or curvilinear in the form task) and the irrelevant feature (angular or curvilinear in the colour task, and green or blue in the form task), for the Control and Active trials separately (eight regressors per block). To account for trial-by-trial variation in reaction time^122^, trials were modelled as events lasting from stimulus onset until response^123–125^ convolved with the haemodynamic response of SPM5.

#### MVPA: ROI analysis

We implemented MVPA using the Decoding Toolbox^126^, which wraps the LIBSVM library^127^. For each participant and ROI (MD regions, stimulated dlPFC, LOC, V4 and early visual cortex), a linear support vector machine was trained to decode colour (green vs. blue) and form (angular vs. curvilinear) when relevant (e.g., angular vs. curvilinear in form task) and irrelevant (e.g., angular vs. curvilinear in colour task) under the two separate TMS conditions (Control or Active), resulting in eight separate classification schemes. In total, there were 16 blocks for each participant (*N* = 20): eight with colour relevant, and eight with form relevant. Since TMS trials were intermingled, half of the trials in these eight blocks contributed to the classification in the Control condition, and half contributed to classification in the Active condition. Each condition (e.g., angular when relevant under Active TMS) consisted of 27.6 trials (correct only—on average across participants) with a SD of 3.2.

For each classification scheme, we used a leave-one-out eightfold splitter whereby the classifier was trained using the data from seven out of the eight blocks and subsequently tested on its accuracy at classifying the unseen data from the remaining block. This procedure was repeated iterating over all possible combinations of training and testing blocks. The accuracies were then averaged over iterations. This was repeated for each classification scheme, participant and ROI separately. We did not use feature selection or dimensionality reduction.

We entered the classification scores for the MD regions into a four-factor ANOVA with factors TMS (Control, Active), Feature (Colour, Form), Relevancy (Relevant, Irrelevant) and Region (left dlPFC, left AI/FO, right AI/FO, ACC/pre-SMA, left IPS and right IPS). For the remaining ROIs (right dlPFC, LOC, V4 and early visual cortex), since they do not form a single network, we conducted separate ANOVAs with factors TMS (Control, Active), Feature (Colour, Form) and Relevancy (Relevant, Irrelevant). Significant interactions were followed up with post hoc analyses. ANOVA main effects and interactions are reported with two-tailed *P* values. We also report 95% confidence intervals and effect size; partial *η*^*2*^ for ANOVA calculated in SPSS (IBM Corp. Released 2016. IBM SPSS Statistics, Version 24.0), and Cohen’s *d* calculated in JASP v0.9^128^. We applied Bayes Factor (BF) analyses using a default uniform prior and Markov chain Monte Carlo settings in JASP v0.9^128^ to interpret all null effects: BF > 3 indicates evidence for experimental hypothesis, and BF < 1/3 indicates evidence for the null hypothesis^129,130^. Where outliers were present (± 3 SD), we performed a log transformation (log10) on the raw classification output prior to conducting ANOVAs and post hoc analyses. This was the case for classification in the MD network.

Classification accuracies were compared against chance, where appropriate, using a two-step permutation test^131^. For this, we exhaustively permuted the class labels for each classification analysis (128 unique combinations). The classifier was trained and tested on each permutation. Following this, we built a group-level null distribution for each condition by sampling (with replacement) 10,000 times from the set of participants x 128 permutation results. For the final step, we calculated the probability (*p*) of the observed classification accuracy given the null distribution, in which *P* = (*k* + 1)/(*n* + 1) where *k* is the number of permutations in the group null with equal or higher accuracy to the actual value and *n* is the number of all permutations in the group null.

#### MVPA: Searchlight analysis

To identify any additional brain regions coding task-related information under Control and Active TMS, we carried out an exploratory classification analysis using a roaming spotlight^62^. In addition to revealing any large effects that our ROI analysis missed, this analysis could potentially be useful to identify candidate ROIs for future studies. For each participant, we extracted data from a spherical ROI (radius 10 mm) centred in turn on each voxel in the brain. A linear support vector machine was trained and tested as before, using data from each sphere, and the classification value for that sphere was assigned to the central voxel yielding whole-brain classification maps for each individual. N/B: The whole-brain searchlight analysis was originally conducted using a sphere of 5-mm radius, but this was later changed to a sphere of 10-mm radius at the reviewer request. The effect on relevant coding at 10 mm was slightly more extensive than for the 5-mm spheres, but in both cases, there was no indication of a release from suppression for irrelevant information.

To combine data across individuals, individual-subject classification accuracy maps were normalised and subsequently smoothed using an 8-mm FWHM Gaussian kernel. In addition to a repeated-measures ANOVA including all experimental conditions (factors: TMS, Feature, Relevancy), mimicking our ROI analyses, paired *t* tests were conducted to directly compare coding between Active and Control TMS under the two relevancy conditions. Classification accuracy was compared to chance at the group level using a one-tailed one-sample *t* test against chance (50%). Since we ran this exploratory analysis to identify additional regions outside of our a priori ROIs where information coding was modulated by TMS, we used a lenient voxelwise threshold of *P* < 0.0001 and corrected for multiple comparisons at the cluster level using FWE (*P* < 0.05). We identified the anatomical locations of significant clusters using the Brodmann and AAL templates of MRICroN^132^ and the Harvard-Cortical and subcortical structural atlases of FSL^133^.

### Reporting summary

Further information on research design is available in the Nature Research Reporting Summary linked to this article.

## Supplementary information

Supplementary Information

Reporting Summary

## Data Availability

The ethical approval for this study does not allow us to share raw data openly. Source data for Figs. 3a–b, 5a–f and 6a–c, and template regions of interest are publicly available on Open Science Framework (https://osf.io/r3g7c/).

## Code availability

The code used to analyse this study is publicly available on Open Science Framework (https://osf.io/r3g7c/).
