# Supplementary material for: Concurrent neuroimaging and neurostimulation reveals a causal role for dlPFC in coding of task-relevant information
Source: Commun Biol. 2021 May 17;4:588. doi: 10.1038/s42003-021-02109-x (PMC8128861; doi:10.1038/s42003-021-02109-x)
Supplement: Supplementary file 1 — Supplementary Information [file 42003_2021_2109_MOESM1_ESM.pdf]

Supplementary Information

Concurrent neuroimaging and neurostimulation reveals a causal role for dlPFC in coding of task-relevant information

Jade B. Jackson[1,2*], Eva Feredoes[3], Anina N. Rich[2], Michael Lindner[3], Alexandra Woolgar[1,2]

[1] *MRC Cognition and Brain Sciences Unit, University of Cambridge, Cambridge, CB2 7EF, UK.*

[2] *Perception in Action Research Centre, Department of Cognitive Science, Macquarie University, Sydney, NSW 2109, Australia.*

[3] *School of Psychology and Clinical Language Sciences, University of Reading, Reading, RG6 6AH, UK.*

**Corresponding author email: jade.jackson@mrc-cbu.cam.ac.uk*

Supplementary Fig. 1: Bar chart representation of TMS effect on accuracy and reaction time data

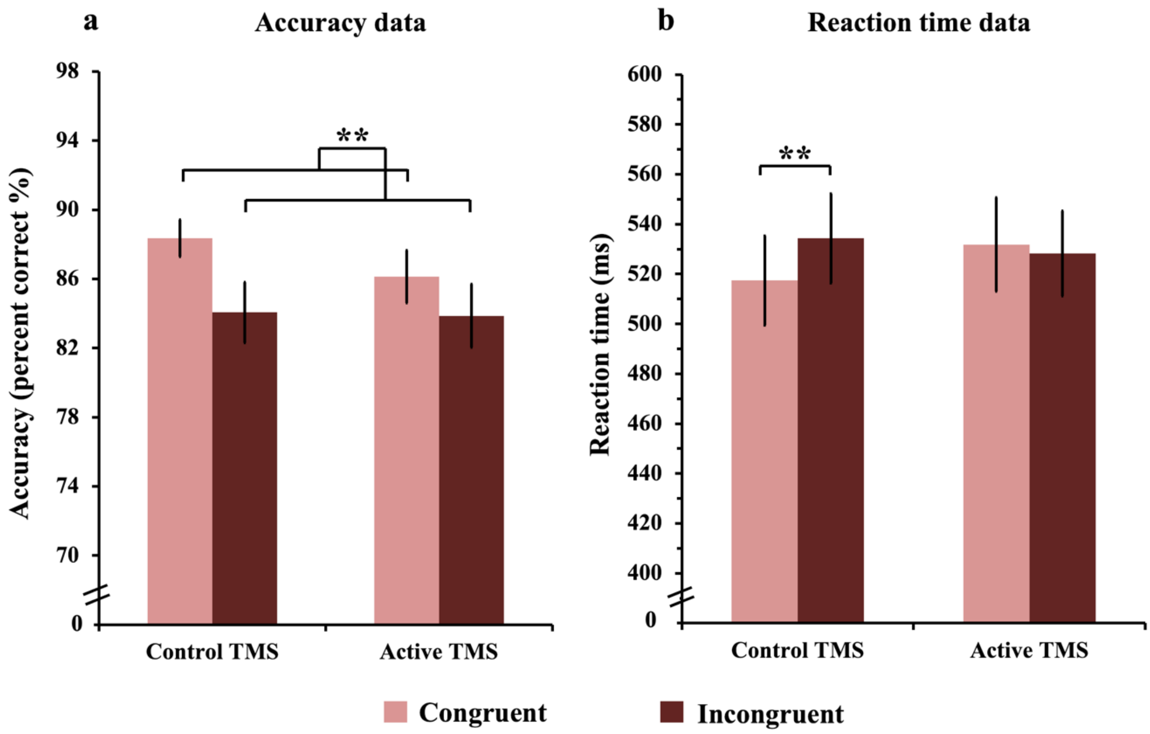

Accuracy data (**a**) showed a main effect of congruency (no interaction). RT data (**b**, correct trials only) revealed that participants were faster in congruent trials than incongruent trials under the Control TMS condition but showed no evidence of a congruency effect under Active TMS (significant interaction). Error bars indicate standard error. Lighter-coloured bars depict congruent trials, and darker-coloured bars depict incongruent trials. **$p < 0.01$. N=20 participants.

Supplementary Fig. 2: Bar chart representation of coding of relevant and irrelevant information in MD regions under Control and Active TMS conditions

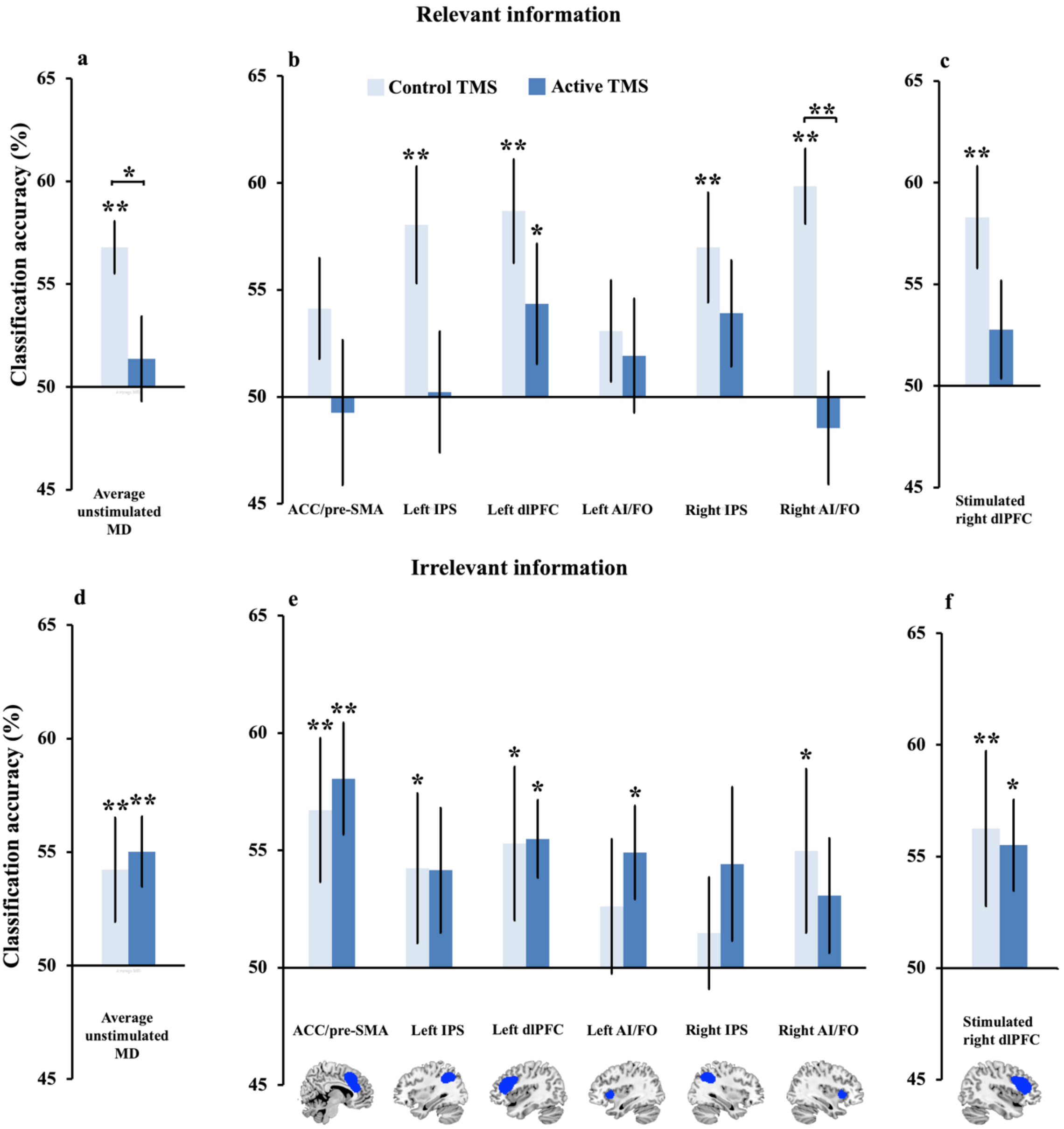

a-c show coding of relevant information (e.g., colour during the colour task) under Control and Active conditions, collapsed across feature (colour, form). **d-f** show coding of irrelevant information (e.g., colour during the form task) under Control and Active conditions, also collapsed across feature. All bars represent coding of identical stimulus information, variation in the strength of coding is driven by TMS intensity and whether the information was relevant for the participant's current task. Due to outliers (>3 SD from the condition mean) we performed a log transformation on the unstimulated MD region data before statistical testing. The data displayed are in the untransformed form prior to log transformation. An ANOVA on the unstimulated MD regions (**a, d**) showed a significant TMS * relevancy interaction. TMS reduced coding of relevant features in unstimulated MD regions, but did not modulate coding of irrelevant information ($BF_{10} = 0.24$). The ANOVA for right dlPFC (factors:

TMS, *Feature*, and *Relevancy*; **c, f**) showed no significant main effects or interactions. Error bars indicate standard error. Lighter-coloured bars depict coding under control TMS, and darker-coloured bars depict coding under Active TMS trials. The significance markings for individual bars indicate whether coding was significantly greater than chance in each condition separately (permutation test). *p<0.05. In this Figure only, ** is equal to p<0.008 (to correct for multiple comparisons in 6 unstimulated MD regions). N=20 participants.

Supplementary Fig. 3: Bar chart representation of coding in visual ROIs under Control and Active TMS

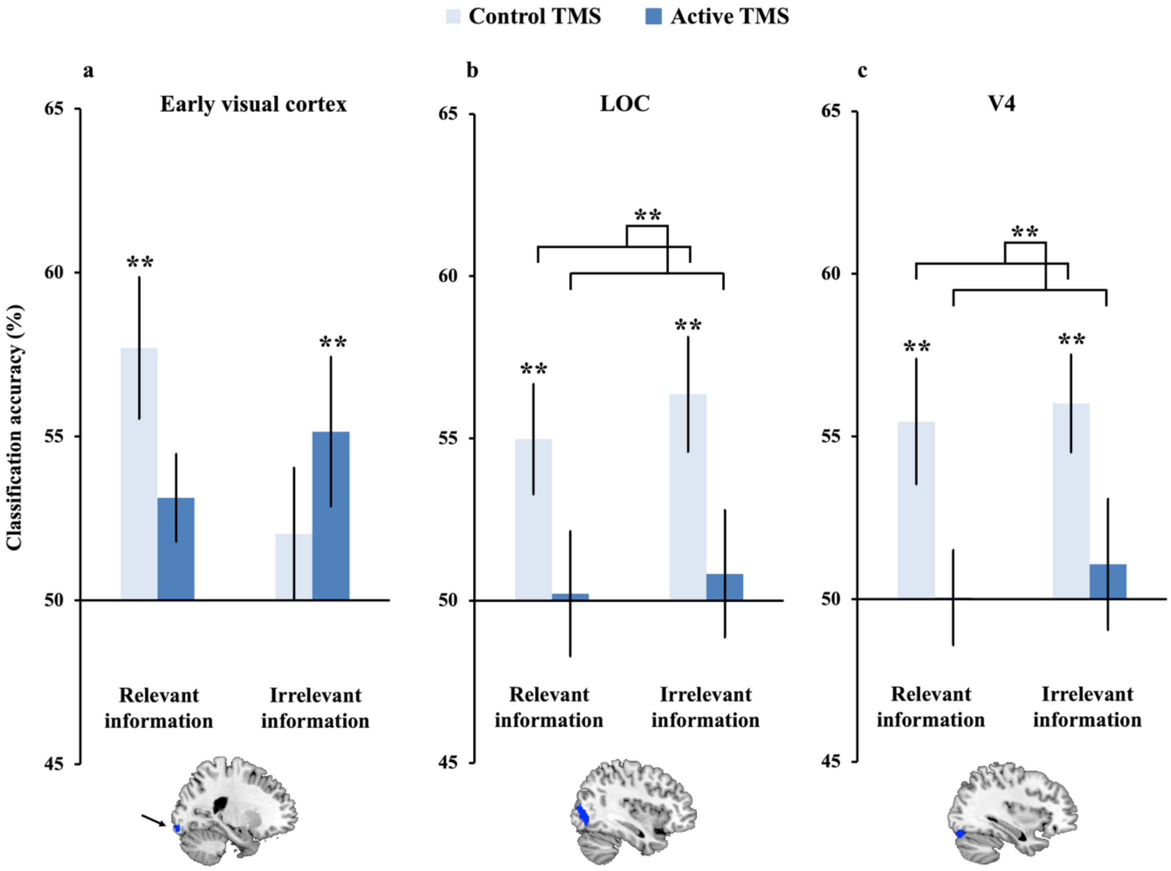

a: Early visual cortex (central visual field). This ROI was derived from individual-participant localiser data and defined as the region stimulated by visual information at fixation (encompassing the same area of central visual field as the objects in the main experimental task) minus visual information outside fixation. There were no significant main effects or interactions. **b:** Lateral Occipital Complex (LOC). This ROI was derived from localiser data as the region more active for viewing of whole objects over scrambled objects. There was stronger coding under the Control TMS condition compared to the Active condition modulated by a Feature*TMS interaction reflecting a stronger effect of TMS on colour than form coding. **c:** V4. This ROI was derived from coordinates from the literature [1] and transformed into native space for each participant. There was again a main effect of TMS modulated by a Feature*TMS interaction reflecting a stronger effect of TMS on colour than form

coding. Error bars indicate standard error. Lighter-coloured bars depict coding under control TMS, and darker-coloured bars depict coding under Active TMS trials. Significance markings for individual bars indicate whether coding was significantly greater than chance in each condition separately (by permutation). *p<0.05; **p<0.01. N=20 participants.

Supplementary Table 1: Peak coordinates for univariate contrast Active > Control TMS. The results were thresholded at $p < 0.0001$ (FWE correction of $p < 0.05$ at cluster level). N=20 participants.

Contrast	Cluster	Hemisphere	Peak coordinates x	y	z	Brodmann area	Cluster size	t	FWE (p)
Univariate (Active > Control)	dorsolateral prefrontal cortex	left	-40	38	10	45	225	7.32	<0.0001
	primary visual cortex extending into extrastriate cortex	right	14	-78	0	17/18	416	7.13	<0.0001
	heschl's gyrus	left	-42	-22	12	48	236	6.83	<0.0001
	superior temporal gyrus	left	-44	2	6	13	42	6.41	=0.046
	anterior cingulate cortex	right	-8	42	18	32	57	6.23	=0.019
	superior temporal gyrus	left	-38	-4	-6	22	197	5.77	<0.0001
	superior temporal gyrus	right	38	-12	-8	22	56	5.75	=0.02
	extrastriate occipital	left	-14	-62	-2	18	61	5.53	=0.015

Supplementary Table 2: Peak coordinates for whole-brain searchlights. Relevant information under Control (upper panel), and irrelevant information under Control TMS (lower panel). The results were thresholded at $p < 0.0001$ (FWE correction of $p < 0.05$ at cluster level). N=20 participants.

Contrast	Cluster	Hemisphere	Peak coordinates x	y	z	Brodmann area	Cluster size	t	FWE (p)
Relevant (Control condition)	peak in precuneus/cingulate gyrus. cluster extends to cerebellum/lingual gyrus/intraparietal sulcus/precentral gyrus/anterior cingulate/anterior insula/middle and inferior frontal gyrus	peak in left but cluster extends bilaterally	-12	-56	8	17	39083	12.8	<0.0001
	precentral gyrus	right	54	6	26	6	468	6.45	=0.002
	supramarginal gyrus	left	-40	-34	28	48	872	6.15	<0.0001
	middle frontal gyrus	right	34	28	20	48	138	5.08	=0.038
Irrelevant (Control condition)	peak in precentral gyrus. cluster extends to precuneus, anterior cingulate, lateral occipital complex, intraparietal sulcus,	peak in right but cluster extends bilaterally	0	-32	60	nearest is 4	7815	8.99	<0.0001

Contrast	Cluster	Hemisphere	x	y	z	Brodmann area	Cluster size	t	FWE (p)
	superior parietal lobule and middle frontal gyrus								
	peak in amygdala. cluster extends to precuneus, hippocampus, insula, putamen and lateral occipital complex	peak in left but cluster extends bilaterally	-20	-4	-24	28	12456	7.16	<0.0001
	frontal orbital cortex	right	30	32	-8	47	172	6.18	=0.03
	paracingulate gyrus	right	8	46	6	32	449	5.73	=0.004
	paracingulate gyrus	right	10	32	30	32	269	5.68	=0.013

Supplementary Table 3: Peak coordinates for whole-brain searchlights. Relevant information under Control > Active (upper panel), and irrelevant information under Control > Active (lower panel). The results were thresholded at $p < 0.0001$ (FWE correction of $p < 0.05$ at cluster level). All significant clusters had regions that were also significantly coded against chance (depicted in Supplementary Table 2, Figure 7). N=20 participants.

Contrast	Cluster	Hemisphere	Peak coordinates			Brodmann area	Cluster size	t	FWE (p)
			x	y	z				
Relevant (Control > Active)	superior frontal gyrus extending towards anterior cingulate	peak in right but cluster extends bilaterally	12	10	64	6	817	8.82	<0.0001
	middle temporal gyrus	left	-62	-28	-18	20	298	7.81	=0.004
	lateral occipital complex extending to precuneus	right	34	-56	40	40	762	6.88	<0.0001
	anterior cingulate gyrus	peak in left but cluster extends bilaterally	-4	20	28	24	835	6.77	<0.0001
	cerebellum	left	-8	-50	-18	19	231	6.39	=0.009
	peak in temporal occipital fusiform. luster extends to cerebellum and parahippocampal gyrus	right	34	-38	-18	37	749	6.12	<0.0001
	peak in lingual gyrus. cluster extends to occipital fusiform and intracalcarine cortex.	bilateral	0	-78	-14	14	308	5.52	=0.004
Irrelevant (Control > Active)	peak in occipital fusiform gyrus. cluster extends to occipital pole and lateral occipital complex	left	-22	-84	-4	18	811	7.09	<0.0001
	peak in temporal fusiform extending into parahippocampal gyrus	left	-40	-16	-20	20	531	6.95	=0.001

thalamus	right	12	-8	-4	nearest is 48	202	6.92	=0.016
occipital fusiform gyrus	left	-40	-58	-16	37	385	6.51	=0.003
cingulate gyrus	left	-4	-6	42	24	204	6.14	=0.016
heschl's gyrus	right	36	-20	16	48	325	5.84	=0.005
lingual gyrus	bilateral	6	-74	-2	17	208	5.77	=0.015
supramarginal gyrus	left	-46	-28	22	48	125	5.7	=0.039
precuneus	right	10	-36	62	nearest is 4	126	5.09	=0.039

Supplementary References

1. Van Leeuwen, T.M., et al., *Color specificity in the human V4 complex: An fMRI repetition suppression study*, in *Advanced brain neuroimaging topics in health and disease-methods and applications*. 2014, Intech. p. 275-295.
