# Supplementary material for: Concurrent neuroimaging and neurostimulation reveals a causal role for dlPFC in coding of task-relevant information
Source: Commun Biol. 2021 May 17;4:588. doi: 10.1038/s42003-021-02109-x (PMC8128861; doi:10.1038/s42003-021-02109-x)
Supplement: Supplementary file 2 — Reporting Summary [file 42003_2021_2109_MOESM2_ESM.pdf]

nature research

Corresponding author(s): Jade B. Jackson

Last updated by author(s): Mar 12, 2021

Reporting Summary

Nature Research wishes to improve the reproducibility of the work that we publish. This form provides structure for consistency and transparency in reporting. For further information on Nature Research policies, see Authors & Referees and the Editorial Policy Checklist .

Statistics

For all statistical analyses, confirm that the following items are present in the figure legend, table legend, main text, or Methods section.

n/a	Confirmed	
☐	☒	The exact sample size (n) for each experimental group/condition, given as a discrete number and unit of measurement
☐	☒	A statement on whether measurements were taken from distinct samples or whether the same sample was measured repeatedly
☐	☒	The statistical test(s) used AND whether they are one- or two-sided<br>*Only common tests should be described solely by name; describe more complex techniques in the Methods section.*
☐	☒	A description of all covariates tested
☐	☒	A description of any assumptions or corrections, such as tests of normality and adjustment for multiple comparisons
☐	☒	A full description of the statistical parameters including central tendency (e.g. means) or other basic estimates (e.g. regression coefficient) AND variation (e.g. standard deviation) or associated estimates of uncertainty (e.g. confidence intervals)
☐	☒	For null hypothesis testing, the test statistic (e.g. F, t, r) with confidence intervals, effect sizes, degrees of freedom and P value noted<br>*Give P values as exact values whenever suitable.*
☐	☒	For Bayesian analysis, information on the choice of priors and Markov chain Monte Carlo settings
☐	☒	For hierarchical and complex designs, identification of the appropriate level for tests and full reporting of outcomes
☐	☒	Estimates of effect sizes (e.g. Cohen's d, Pearson's r), indicating how they were calculated

Our web collection on statistics for biologists contains articles on many of the points above.

Software and code

Policy information about availability of computer code

Data collection	Data were collected using the Psychophysics Toolbox version-3 software in MATLAB, as described in the methods
Data analysis	Univariate analyses were carried out using SPM5, multivariate decoding analyses using The Decoding Toolbox, and statistical analyses using SPSS and JASP, as described in the methods

For manuscripts utilizing custom algorithms or software that are central to the research but not yet described in published literature, software must be made available to editors/reviewers. We strongly encourage code deposition in a community repository (e.g. GitHub). See the Nature Research guidelines for submitting code & software for further information.

Data

Policy information about availability of data

All manuscripts must include a data availability statement. This statement should provide the following information, where applicable:

- Accession codes, unique identifiers, or web links for publicly available datasets
- A list of figures that have associated raw data
- A description of any restrictions on data availability

The ethical approval for this study does not allow us to share raw data openly. Source data for Figures 3, 5 and 6, template regions of interest, and code used to analyse this data are publicly available on Open Science Framework (https://osf.io/r3g7c/).

Field-specific reporting

Please select the one below that is the best fit for your research. If you are not sure, read the appropriate sections before making your selection.

Life sciences study design

All studies must disclose on these points even when the disclosure is negative.

Sample size	A sample size of 20 participants was chosen based on previous literature using similar paradigms, and was the maximum possible given the available funding, the requirement to scan each participant twice, and the technical challenges involved in acquiring fMRI data concurrent with TMS. A post hoc power analysis estimated that for our main analysis of interest we achieved 62% power to detect a TMS*Relevancy interaction at alpha = 0.05 for the reported effect size of partial eta-squared = 0.2.
Data exclusions	Thirty-one healthy volunteers signed up for the experiment. However, four participants did not pass the TMS screening requirements, and seven participants did not complete the second scanning session so the data from these 11 participants are not included.
Replication	These findings have not yet been replicated. TMS-fMRI is still unusual and technically and practically challenging to carry out.
Randomization	There was no randomization as this is a repeated measures (within-subjects) design
Blinding	Blinding was not relevant to this study as it was a repeated measures (within-subjects) design

Reporting for specific materials, systems and methods

We require information from authors about some types of materials, experimental systems and methods used in many studies. Here, indicate whether each material, system or method listed is relevant to your study. If you are not sure if a list item applies to your research, read the appropriate section before selecting a response.

Materials & experimental systems

n/a	Involved in the study	
x		Antibodies
x		Eukaryotic cell lines
x		Palaeontology
x		Animals and other organisms
	x	Human research participants
x		Clinical data

Methods

n/a	Involved in the study	
x		ChIP-seq
x		Flow cytometry
	x	MRI-based neuroimaging

Human research participants

Policy information about studies involving human research participants

Population characteristics	The final group consisted of twenty participants (15 female, 5 male; mean age = 21.6 years, SD= 3.36). All participants were right-handed with normal or corrected-to-normal vision and no history of neurological or psychiatric disorder.
Recruitment	Participants were recruited through the University of Reading's School of Psychology Research Panel (online advertisement). Accordingly the sample is biased in that it consisted solely of undergraduate or postgraduate students at the University of Reading.
Ethics oversight	The experiment was approved by the University of Reading Research Ethics Committee.

Note that full information on the approval of the study protocol must also be provided in the manuscript.

Magnetic resonance imaging

Experimental design

Design type	Event-related
Design specifications	In the experimental session (session 2) participants completed 8 runs of trials, each of which consisted of two blocks, one of the color task and one of the form task, for a total of 1536 trials, as specified in the Methods.
Behavioral performance measures	We recorded button presses which we analysed for reaction time and accuracy. As specified in the methods, we compared behavioural data for stimuli in which colour and form mapped onto the same button-press response (congruent) to those where the two stimulus dimensions indicated different button-press responses (incongruent), using three-way repeated measures ANOVAs, with factors TMS (Control, Active), Feature (Colour, Form), and Congruency (Congruent, Incongruent), with post-hoc t-tests. We report means, confidence intervals, effect size and probability.

Acquisition

Imaging type(s)

functional

Field strength

3T

Sequence & imaging parameters

We used a sequential ascending T2*- weighted EPI acquisition sequence with the following parameters: acquisition time 2450ms; echo time 30 ms; 35 oblique axial slices with a slice thickness of 3.0 mm and a 0.70 mm inter-slice gap; in plane resolution 3.0×3.0 mm; matrix 64×64; field of view 256 mm; flip angle 90°; 50% phase oversampling in the phase encoding direction to shift any Nyquist ghost artefact, due to the presence of the TMS coil, to outside the volume of interest.

Area of acquisition

Whole brain

Diffusion MRI ☐ Used ☒ Not used

Preprocessing

Preprocessing software

MRI data were preprocessed using SPM 5 (Wellcome Department of Imaging Neuroscience, www. fil.ion.ucl.ac.uk/spm) in MatLab 2013b. Functional MRI data were converted from DICOM to NIFTI format, spatially realigned to the first functional scan and slice timing corrected, and structural images were co-registered to the mean EPI. EPIs were smoothed slightly (4 mm FWHM Gaussian kernel) to improve signal-to-noise ratio for multivariate analyses, and were smoothed separately with a larger smoothing kernel for univariate analyses (8 mm FWHM Gaussian kernel). In all cases the data were high pass filtered (128s).

Normalization

EPI data for multivariate analysis were not normalised and were analysed in native space. Structural scans were normalised, using the linear segment and normalise routine of SPM5 (Wellcome Department of Imaging Neuroscience, London, UK; www.fil.ion.ucl.ac.uk) to derive the individual participant normalisation parameters needed for transformation of ROIs into native space, TMS target definition, and to normalise the searchlight accuracy maps derived in native space.

Normalization template

T1 template of SPM5

Noise and artifact removal

We removed artefacts associated with the TMS pulse only. For this, we first identified slices with a signal magnitude of > 1.5 SD from the run mean and visually inspected them visually for presence of the TMS artefact. These slices were replaced by the mean of the same slices from the preceding and proceeding volumes (following Feredoes et al., 2011). Next, we manually removed and interpolated over any remaining slices that were acquired during TMS pulse delivery, identifying them based on timing and visual inspection. This was necessary because, depending on the affected slice, the Control TMS condition did not always produce deviations > 1.5SD from the mean. We ensured that the same number of slices were removed and interpolated over in the Active and Control TMS conditions.

Volume censoring

We did not censor any volumes

Statistical modeling & inference

Model type and settings

We carried out both mass univariate and multivariate tests as detailed in the Methods. For the main analysis, we specified a first level design in which we estimated the activity associated with the two colours and two forms of the objects, using correct trials only. Each trial contributed to the estimation of two beta values: the relevant feature (green or blue in the colour task, and cuby or smoothy in the form task) and the irrelevant feature (cuby or smoothy in the colour task, and green or blue in the form task), for the Control and Active trials separately (8 regressors per block). To account for trial by trial variation in reaction time (Todd, Nystrom, & Cohen, 2013), trials were modelled as events lasting from stimulus onset until response (Henson, 2007; Grinband, Wager, Lindquist, Ferrera, & Hirsch, 2008; Woolgar, Golland, & Bode, 2014) convolved with the hemodynamic response of SPM5. Second level analyses are random effects (across subjects).

Effect(s) tested

For the main analysis, we entered classification scores for the MD regions into a four factor ANOVA with factors TMS (Control, Active), Feature (Colour, Form), Relevancy (Relevant, Irrelevant) and Region (Left dlPFC, Left AI/FO, Right AI/FO, ACC/pre-SMA, Left IPS, Right IPS). For the remaining ROIs (right dlPFC, LOC, V4, early visual cortex), since they do not form a single network, we conducted separate ANOVAs with factors TMS (Control, Active), Feature (Colour, Form), Relevancy (Relevant, Irrelevant). Significant interactions were followed up with post hoc analyses and permutation tests to compare classification accuracy to chance where appropriate.

Specify type of analysis: ☐ Whole brain ☐ ROI-based ☒ Both

Anatomical location(s)

We derived labels manually using the Brodmann and AAL templates of MRICroN and the Harvard-Cortical and subcortical structural atlases of Fsl

Statistic type for inference
(See Eklund et al. 2016)

Our main inferences are made on an ROI basis. Additional inference at whole brain level comes from an exploratory searchligh-based whole brain analysis of classification, which was assessed for inference at the cluster level. Since we ran this exploratory analysis to check for the specificity of the results (i.e. to rule out that it was a very general effect across many brain regions) we used a lenient voxelwise threshold of $p < 0.0001$ and corrected for multiple comparisons at the cluster-level using FWE.

Correction | FWE

Models & analysis

n/a	Involved in the study
☒	☐ Functional and/or effective connectivity
☒	☐ Graph analysis
☐	☒ Multivariate modeling or predictive analysis

Multivariate modeling and predictive analysis

For each participant and ROI (MD regions, stimulated dlPFC, LOC, V4, early visual cortex), a linear support vector machine was trained to decode colour (green vs. blue) and form (cuby vs. smoothy) when relevant (e.g., cuby vs. smoothy in form task) and irrelevant (e.g., cuby vs. smoothy in colour task) under the two separate TMS conditions (Control or Active) resulting in 8 separate classification schemes. In total, there were 16 blocks for each participant: 8 with colour relevant, and 8 with form relevant. Since TMS trials were intermingled, half of the trials in these 8 blocks contributed to the classification in the Control condition, and half contributed to classification in the Active condition.

For each classification scheme, we used a leave-one-out 8-fold splitter whereby the classifier was trained using the data from 7 out of the 8 blocks and subsequently tested on its accuracy at classifying the unseen data from the remaining block. This procedure was repeated iterating over all possible combinations of training and testing blocks. The accuracies were then averaged over iterations. This was repeated for each classification scheme, participant and ROI separately. We did not use dimensionality reduction or feature selection. Data were evaluated using ANOVA as specified above and in the Methods.
